# From Solvent-Mediated Micellization to Packing in a Face-Centered Cubic Structure of Poloxamers

**DOI:** 10.1021/acs.macromol.6c00316

**Published:** 2026-06-09

**Authors:** Seyed Mostafa Tabatabaei, Javen S. Weston, John Klier, Reza Foudazi

**Affiliations:** † School of Sustainable Chemical, Biological and Materials Engineering, 535706 The University of Oklahoma, Norman, Oklahoma 73019, United States; ‡ Chemical Engineering Department, University of Tulsa, Tulsa, Oklahoma 74104, United States

## Abstract

Ethylammonium nitrate (EAN) is one of the most studied protic ionic liquids (ILs) offering unique properties for self-assembly of poloxamers. Understanding how solvent composition in EAN–water mixtures governs block copolymer self-assembly has yet to be investigated in full detail. This study systematically investigates the temperature-driven self-assembly of Poloxamer 407 in the full composition range of EAN–water mixtures, combining small-angle X-ray scattering (SAXS), rheology, and differential scanning calorimetry (DSC) to resolve micellization and lyotropic liquid crystal (LLC) formation, also termed gelation in the literature. This work reveals a distinct nonmonotonic dependence of both micellization and LLC transitions on EAN content in the solvent, where a minimum at 60:40 wt % EAN:water ratio is observed. We identify a universal trend in the evolution of micelle volume fraction against normalized temperature independent of solvent nature and surfactant concentration. Extrapolation of this sigmoidal trend shows a critical micelle volume fraction of approximately 0.49 required for face-centered cubic (FCC) ordering, which remains invariant across all solvent environments and agrees with the entropic bonding theory of colloidal crystallization. Moreover, we resolve two temperature-dependent micellization regimes in the transition region, characterized by a decrease in micellar number density at early stages, followed by micellization and micelle growth through unimer incorporation, resulting in an increase in number density.

## Graphical abstract

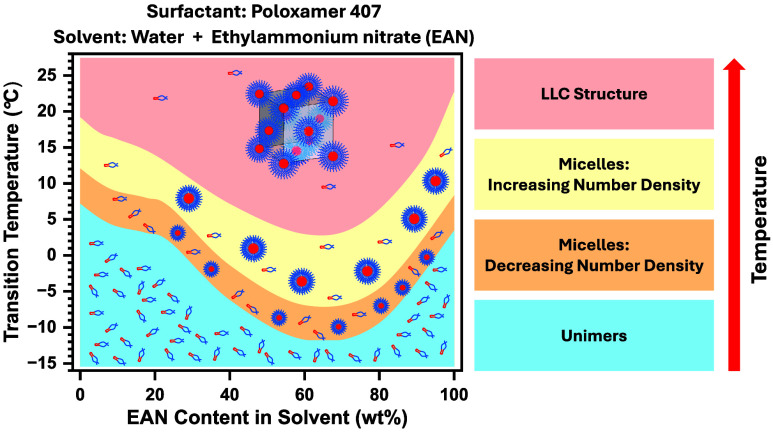

## Introduction

LLCs have become indispensable platforms for the design of advanced functional materials with broad applications spanning membrane separation,^1,2^ biomedical devices,^3,4^ catalysis,^5,6^ drug delivery,^7,8^ and energy storage.^9^ Their characteristic nanostructural order, coupled with highly tunable physicochemical properties, makes them ideal for engineering materials with precisely defined pore structures and transport pathways.^10^ LLCs are formed via self-assembly of amphiphilic molecules in selective solvents, yielding mesophases with long-range periodic order at the nanometer scale, typically between 2 and 50 nm.^11^ Typical LLC morphologies include lamellar arrangements of planar micelles, hexagonal arrays of rod-like micelles, and cubic packing of spherical micelles, such as FCC and body-centered cubic (BCC) lattices and bicontinuous cubic phases.^12^ The formation of these structures depends on the geometry and packing of the amphiphilic molecules, and the resulting mesophase is highly sensitive to parameters such as the concentration, temperature, and solvent-amphiphile interactions.

Poloxamers, amphiphilic triblock copolymers of poly­(ethylene oxide)–poly­(propylene oxide)–poly­(ethylene oxide), PEO-PPO-PEO, are widely used in LLC systems. These polymers, also known as Pluronics as their commercial trademark developed by BASF, form micelles in water with a lipophilic phase made of almost dry PPO and a hydrophilic phase made of solvated PEO.^13–15^ Their versatility stems from a broad range of molecular weights and variable PEO:PPO ratios, which allow fine-tuning of the micelle shape, aggregation behavior, and mesophase structure.^16^ This diversity gives rise to complex phase diagrams, enabling the formation of various LLC structures under different conditions.^17^ Detailed structural properties of different poloxamers along with their equivalent Pluronic names have been reported in the literature.^18^

As structure-directing agents, poloxamers play a pivotal role in directing the nanostructure and responsiveness of LLC-templated materials. Commonly used poloxamers in LLC systems include, but are not limited to, Poloxamer 407 (P407),^19,20^ P188,^21,22^ and P234.^23,24^ In particular, P407 (also known as Pluronic F127) and P188 (also known as Pluronic F68) are frequently employed in biomedical applications due to their FDA approval, excellent biocompatibility, and ability to form well-defined nanostructures suitable for drug delivery, tissue engineering, and diagnostic platforms.^25^ Poloxamers are also used in many everyday-life materials, such as detergents and coatings, due to their tunable adsorption, self-assembly, and surface-modifying properties at solid–liquid interfaces.^26^

Water has been conventionally used as a common solvent to mix with poloxamers for obtaining various mesophases. Water promotes endothermic and entropically driven micellization of poloxamers through dehydration of the PPO segment while forming a strong hydrogen bonding between PEO blocks and water.^27^ ILs have been considered as a replacement for water in many applications, including self-assembly of amphiphilic materials. They are salts in the liquid state at room temperature that are composed of organic cations and organic or inorganic anions.^28^ They have unique properties such as negligible vapor pressure, high density, low flammability, and high ion conductivity.^29^ Although amphiphilic block copolymers show lower solvophobicity in ILs due to their higher solubility of hydrocarbons compared to that of water, similar self-assembled structures have also been reported in selective ILs.^30^ However, this lower solvophobicity results in higher critical micellization concentration (CMC), meaning that usually a higher concentration of amphiphile is required to achieve the same structures.^31^

Both aprotic^32^ and protic^33^ ILs have shown entropy-driven micellization similar to that of water. However, the application of ILs in self-assembly is beyond the promotion of micellization. ILs have been considered for LLC templating to avoid polymerization-induced phase separation due to their high viscosity, which hinders diffusion of surfactants during polymerization.^12^ The conductive nature of ILs makes it possible to use LLC-based ionogels in sensing applications.^34^ It has also been reported that soft nanoconfinement of ILs in LLC systems can increase the CO_2_ solubility, absorption capacity, and absorption rate.^35^ These examples illustrate the critical role that ILs play in the formation, properties, and applications of LLC systems.

EAN with a chemical structure of [CH_3_CH_2_NH_3_]^+^[NO_3_]^−^ is reported to be the first room-temperature IL.^36^ PEO has lower solubility in EAN compared to water based on the more compact formation observed by lower radius of gyration.^37^ The ethyl group on the EAN cation promotes the development of distinct polar and nonpolar regions, enhancing the solubility of hydrocarbons. However, its ability to form a hydrogen bonding network similar to water makes it a good replacement for water in micellization of poloxamers.^38^ Despite these similarities, there are some structural differences in the self-assembled micelles. While the core size of the micelles is almost the same, the shell of the spherical micelles of P403 in EAN is 30–40% smaller than that of water due to lower solubility of PEO and dehydration of the shell.^39^ Zhang et al.^40^ studied the phase diagrams of P403 in water and EAN showing similar trends with the formation of micellar cubic, hexagonal, and lamellar phases as P403 concentration increases. Both water and EAN promote P403 self-assembly by solvating the hydrophilic PEO blocks through hydrogen bonding. The phase progression from spherical micelles to cylindrical and then to planar structures is consistent in both systems. However, a key difference is that EAN supports an additional reverse bicontinuous cubic phase, which is absent in water, likely due to EAN’s higher affinity for the hydrophobic PPO blocks.

It is well-known that addition of EAN as a cosolvent to water can promote micellization of poloxamers by decreasing the critical micellization temperature (CMT), *T*_mic_,^41^ as well as the CMC.^42^ However, there are limited studies to the knowledge of the authors, spanning the whole concentration range of EAN:water mixtures for self-assembly. Tsoutsoura et al.^43,44^ studied the self-assembly of P335 in solvents with various EAN:water ratios. They found that increasing the ratio of EAN in the EAN:water mixture promotes significant changes in the self-assembly behavior and structural features of LLCs. In lower EAN concentrations, water dominates the solvation of the PEO blocks, favoring solvated micellar structures. As the EAN concentration increases, it acts as a selective solvent for the PEO blocks, resulting in reduced hydration, changes in the microdomain organization, and altered lattice parameters in hexagonal LLC phases. For example, higher EAN content generally leads to smaller interdomain distances and more compact self-assembled structures, as indicated by decreasing lattice spacings. These effects are linked to EAN’s distribution within the hydrophilic domains, which replaces some of the water to solvate PEO, thereby modulating the overall self-assembly environment of P335 and resulting in tunable hexagonal structures across a wide range of EAN:water compositions.

In this study, we present a comprehensive characterization of LLC phases formed by the self-assembly of Poloxamer 407 in mixtures of EAN and water by systematically varying the EAN content. A combination of rheology, SAXS, and DSC techniques was employed to investigate the structural evolution of P407 across different temperature ranges, capturing transitions from unimers to micelles and eventually to LLC mesostructures. We reveal a nonmonotonic dependence of both gelation and critical micellization temperature with a pronounced minimum at 60:40 wt % EAN:water ratio. Despite strong solvent-dependent changes in micelle structure and transition temperatures, face-centered cubic (FCC) ordering consistently emerges when the micelle volume fraction reaches a solvent-independent threshold of ∼0.49, demonstrating that spatial packing constraints govern LLC formation following a sigmoidal trend, independent of surfactant concentration. Our findings provide a unified mechanistic framework for understanding and tuning poloxamer micellization and self-assembly.

## Experimental Section

### Materials

All materials in this study were used as received unless noted otherwise. The surfactant used in preparing the LLC samples was P407 (PEO_100_-PPO_65_-PEO_100_) with an average molecular weight of approximately 12600 g/mol, which was purchased from Sigma-Aldrich. EAN (>97% purity containing ∼1.4 wt % water, obtained from IoLiTec) and deionized (DI) water with a conductivity of 0.055 μS/cm (EMD Millipore Direct-Q3) were used as solvents to prepare the mesophases.

### Mesophase Preparation

P407 has shown self-assembled FCC structures in both EAN^45^ and water^46^ in the range of 20–30 wt %. In this study, 24 wt % of P407 was used for mesophase preparation since the structure and flow behavior of 24 wt % P407 in EAN have been studied previously and shown potential for different applications.^34,45,47^ Different ratios of EAN and water were used to prepare the solvent phase. In this study, we use *PXEAN* for sample coding, which represents samples in which *X* wt % of the solvent is made of EAN and the rest is water, while the P407 concentration is the same in all samples (i.e., 24 wt %). For instance, 60 wt % of solvent in P60EAN is formed of EAN. Table S1 summarizes all the samples. P407 and the solvent were added to centrifuge tubes and mixed by consecutive hand mixing and centrifugation at 10,000 rpm for 5 min (each cycle) until a clear transparent gel was obtained. None of the samples showed any birefringence under cross-polarized light microscopy (CPLM), which is one of the signs of a cubic structure. In order to investigate the effect of surfactant concentration, samples with 20 and 22 wt % P407 have also been prepared with the same protocol.

### Differential Scanning Calorimetry (DSC)

Due to the thermodynamic nature of gelation in LLC systems, DSC experiments were performed using a TA Instruments DSC2500 calibrated only by heating. Approximately 10 mg of the sample was loaded into aluminum pans (PerkinElmer, Inc.) and sealed hermetically with hermetic lids. Since samples with high water content could crystallize below 0 °C, the lower and upper limits of the thermal analysis were different among samples. A heating/cooling ramp of 2 °C/*min* was chosen to keep the experimental conditions consistent with rheological measurements. The samples were isothermally maintained at the lower and upper limits at the end of each heating/cooling cycle for 5 min, and the experiments were repeated using the same heating/cooling cycle to eliminate the effect of thermal hysteresis.

### Rheology

A strain-controlled ARES-G2 rheometer (TA Instruments) was used to perform all rheological measurements. A concentric cylinder geometry with a moving recessed bottom bob having an outer diameter of 25 mm and a fixed outer cup having an inner diameter of 27 mm was used to better capture the sol state of the samples at low temperatures with low viscosities. For the sol state, the steady flow sweeps were performed in the shear rate range of 0.1 < γ̇ < 30 s^–1^. A specific protocol for temperature ramp tests was developed to determine the sol–gel transition temperature from the crossover of the loss modulus by the storage modulus. A heating/cooling ramp of 2 °C/min was used to maintain a quasi-steady state and ensure to eliminate the effect of temperature ramp on observing sol–gel transition. Considering dynamic moduli is frequency dependent and determining sol–gel transition temperature could be affected by this fact,^48^ our preliminary experiments showed that consistent results with DSC are obtained from temperature ramp tests at the fixed frequency of ω = 40 rad/s, at which the lowest noise to signal ratio was observed. It should be noted that strain amplitudes of 10% and 0.1% were used to perform these measurements in the sol and gel states, respectively. The reason for this specific measurement protocol is explained in the discussion around Figure S1 and Figure 2.

### Small Angle X-ray Scattering (SAXS)

SAXS was used to confirm the FCC structure of the samples and to measure the domain size, core radius, and shell thickness of the spherical micelles. For sample preparation, mesophases were cooled to turn into sol with a low viscosity and loaded into quartz capillary tubes with a nominal diameter of 1.5 mm (Charles Supper Company, Natick, MA) using a long needle. All tubes were sealed properly using an epoxy glue afterward to prevent evaporation when exposed to the vacuum chamber during the measurements. 2D scattering patterns were acquired using a Xenocs XEUSS 3.0 with Cu-K-alpha (1.54 Å) radiation. The results were azimuthally averaged to obtain 1D scattering profiles using the Data Reduction protocols developed by Xenocs and found in the XSACT software package. The apparatus was operated at 50 kV and 0.6 mA with a sample-to-detector distance of 55 cm (corresponding to a *q*-range of 0.01 to 0.3 Å^–1^), absolute pressure in the X-ray flight path of <0.2 mbar, and an exposure time of 20 min. Measurements were made using the ‘High Resolution’ collimation settings to minimize the effect of instrumental smearing. The Peltier stage was used to control the temperature of the samples, while the ramp was chosen to be the same as that for the rheological measurements. A resting time of 5 min before measurements was allowed to ensure that the sample was at equilibrium.

SasView^49^ has been used to fit scattering from micellar solutions using the core–shell sphere form factor and hard sphere structure factor. Scattering from LLC structures has been fitted using an FCC lattice model with paracrystalline distortion. A detailed fitting procedure is presented in the Supporting Information (SI).

## Results and Discussion

Figure 1a presents the SAXS intensity (*I*) versus the scattering vector (*q*) at 45 °C, where all the samples are in the gel state. Based on their isotropic nature observed in CPLM images appearing uniformly black, the mesophases are expected to exhibit a cubic structure. The scattering data reveal Bragg peak ratios of $\sqrt{3}:\sqrt{4}:\sqrt{8}:\sqrt{11}$ , corresponding to the Miller indices (111), (200), (220), and (311), respectively. These ratios, observed for all samples containing EAN, indicate an *Fm*3*m* space group with FCC arrangement of spherical micelles. Peaks at higher [*hkl*] values are weaker due to diminishing long-range order and/or polycrystalline nature of LLCs.

**Figure 1:**
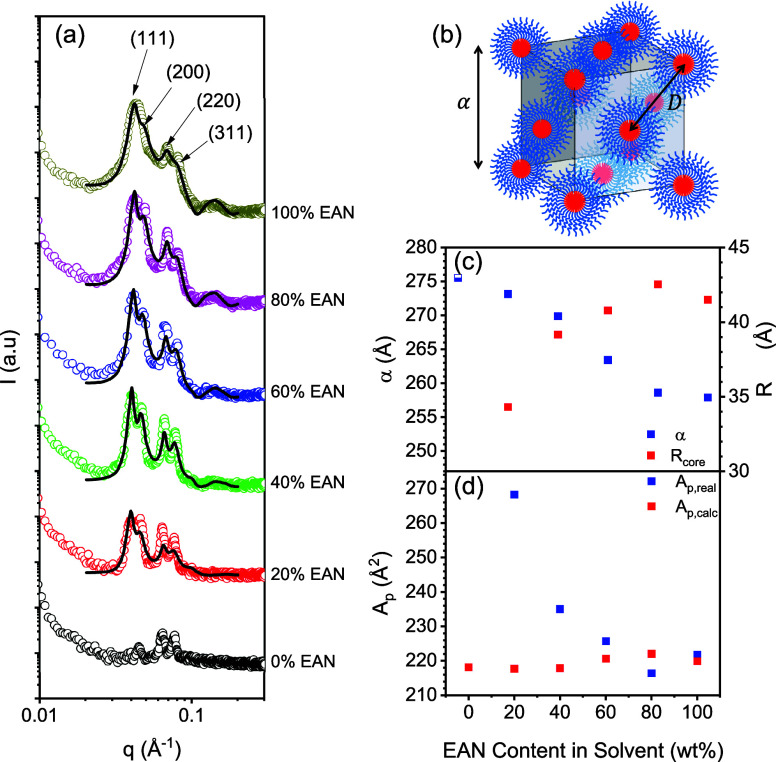
(a) SAXS scattering pattern of samples with different EAN content at 45 °C; empty symbols show the scattering data from SAXS and black lines show FCC model fitting results using eqs S6–S11, (b) schematic of FCC packing of core and shell spherical micelles, and (c) variations of lattice parameter and interfacial area per PEO block in samples with different EAN contents, calculated based on both geometrical parameters obtained from fitting, *A*_*p*,*real*_, and theoretical geometrical parameters according to literature.^54^

Although in the P0EAN mesophase, where water is the only solvent, the first peak (111) is not observed, the ratios of subsequent peaks confirm an FCC structure. Literature reports indicate that the self-assembly of commercially available P407 in water at concentrations similar to this study also results in spherical micelles arranged in an FCC structure.^46,50,51^ However, the removal of diblock impurities^52^ or introducing additives such as salts^53^ can induce phase transitions to body centered cubic (BCC) or simple cubic (SC) structures. The self-assembly of P407 in EAN has also been shown to result in a FCC structure, with a lattice parameter, α, of 28 nm (representing the cubic unit cell size).^45^ In a cubic unit cell, the lattice parameter can be determined using eq 1.^54^

1 $$\alpha =\frac{2\pi \sqrt{h^{2}+k^{2}+l^{2}}}{q_{hlk}}$$

where *h*, *k*, and *l* represent Miller indices, and *q*_*hlk*_ is the scattering vector of the [*hkl*] plane. The lattice parameter can be determined by plotting $\sqrt{h^{2}+k^{2}+l^{2}}$ versus *q*_*hlk*_/2π, where the slope corresponds to α. Scattering data in Figure 1a are fitted to a FCC model with paracrystalline distortion giving the diameter of spheres and nearest neighbor distance, *D*, which correlates to α by α = *D*√2.

It is well-known that the spherical micelles of poloxamers consist of a hydrophobic core, primarily composed of PPO, and a hydrophilic solvated shell made of PEO. Figure 1b illustrates the FCC structure, comprising core–shell spherical micelles, providing a geometrical representation of α and *D*. As shown in Figure 1c, the lattice parameter decreases with increasing EAN fraction in the solvent in the range of 275–256 Å. The radius of sphere obtained from the FCC model may be interpreted as the radius of the spherical micelles in this system. However, as will be discussed later in this section, the range of the radius obtained is within the range of the radius of P407 micelle core. Since the PEO shell is solvated, the electron density difference between shell and the solvent is negligible. However, PPO is nearly insoluble in water^55^ and its solubility in EAN is limited to approximately 1 wt %.^37^ Thus, it is reasonable to assume that the PPO block predominantly forms the solvophobic portion of the lattice. Therefore, it is hypothesized that the model just captures the micelle cores as hard spheres placed in the FCC lattice with the obtained radius of spheres being the core radius. Furthermore, this model uses simple spherical form factor which has been used to fit on the high-*q* data to obtain the core radius, further confirming our interpretation here.^56^

It is important to note that dry core assumption is no longer valid when an oil phase is present in the system. In such cases, the volume fraction of the oil phase must be included in the contribution of the PPO block.^13,57,58^ In the absence of an oil phase, eq 2 is used to calculate the apolar volume fraction, *f*, of the system:^54^

2 $$f=0.332\varphi_{p}$$

Here, φ_*p*_ represents the volume fraction of the polymer in the system, and 0.332 corresponds to the volume fraction of PPO per P407 molecule. The interfacial area per PEO block, *A*_*p*_, can then be calculated using eq 3 as follows:

3 $$A_{p,\mathrm{geometrical}}={\left(36\pi n_{u}f^{2}\right)}^{1/3}\frac{v_{p}}{2\varphi_{p}\alpha }$$

where *n*_*u*_ represents the number of micelles in a single unit cell (i.e., equal to 4 for the FCC structure) and *v*_*p*_ denotes the volume of one P407 block copolymer, approximately 2 × 10^4^ Å^3^.^13^ This simplified equation for calculation of *A*_*p*_ based on the geometry has been frequently used in the literature. However, *A*_*p*_ can be directly calculated using the core radius of the micelles. *N*_agg_, indicating the number of surfactant unimers in one micelle, can be calculated assuming the core is dry and mostly made of PPO chains. Therefore, by dividing the volume of the core, *V*_core_, by the volume of one PPO block in a P407 molecule, *v*_PPO_, *N*_agg_ can be calculated using eq 4:

4 $$N_{\mathrm{agg}}=\frac{V_{\mathrm{core}}}{v_{\mathrm{PPO}}}=\frac{4}{3}\pi R_{c}^{3}\frac{\rho_{\mathrm{PPO}}N_{A}}{M_{w,\mathrm{PPO}}}$$

where ρ_PPO_ is the bulk density of PPO, *N*_A_ is the Avogadro’s number, and *M*_w,PPO_ is the molecular weight of the middle PPO block forming the dry core. Since there are two PEO blocks in each P407 molecule, *A*_*p*_ can be calculated as follows:

5 $$A_{p,\mathrm{fitting}}=\frac{4\pi R_{c}^{2}}{2N_{\mathrm{agg}}}$$

Figure 1d shows the *A*_*p*_ obtained from eqs 3 and 5, representing the values calculated based on simplified geometrical assumptions and values obtained from FCC paracrystalline model fittings, respectively. At first glance, this plot shows that eq 3 is a good approximation to calculate *A*_*p*_ with an average error of about 6%, where no detailed information about the structure of micelles is present. This plot shows that *A*_*p*,geometrical_ has a general increasing trend, while *A*_*p*,fitting_ has a general decreasing trend. The equilibrium area per block copolymer is determined by a balance of the competing forces. The interfacial free energy at the polar/apolar boundary promotes a reduction in surface area, while decreasing the area causes the polymer chains to stretch against the conformational entropy. Additionally, steric repulsion between neighboring PEO brushes also contributes to chain conformation.^54^

In a highly selective solvent, domain spacing in microstructures enlarges as the interfacial area between block domains decreases to minimize solvophobic interactions with the solvent.^43^ Considering the decrease in the lattice parameter, this might be a good reason behind the slight increase in *A*_*p*,geometrical_, which is defined assuming a single hydrophilic solvent in the system. A similar trend has been observed in the case of P403 between lattice parameter of the hexagonal structure and calculated interfacial area with respect to changing EAN fraction in the solvent.^44^ Although the IL may have surface active properties to some extent, the main assumption in this case was that the IL only contributes to the solvophilic–solvophobic interface by swelling the PEO shell. It was concluded that EAN only constitutes ∼2% of the total interfacial volume, which suggests negligible surface activity of the EAN. However, the observed trend in *A*_*p*,geometrical_ shows that more complex interactions at the solvophilic–solvophobic interface play a role, implying a systematic study is needed to investigate the competing forces at the interface.

The SAXS study by Chen et al. suggested that replacing water with EAN has no significant effect on the core radius of P403, and the core radius is slightly higher in the case of water.^39^ However, another study that used small-angle neutron scattering (SANS) reported a slightly higher core radius of the same poloxamer upon addition of EAN.^59^ Higher accuracy of SANS studies through contrast matching makes the latter results more reliable. Although the dry core assumption is always in good agreement with geometrical studies with SAXS, the limited contribution of both solvent and PEO to the core can also be quantified by SANS.^59^ This assumption has also been used and verified for different ILs including EAN.^43,59^ However, as mentioned earlier, the solubility of PPO in EAN is approximately 1 wt %. Thus, slight penetration of solvent into the core cannot be ruled out. Applying the assumption of solvent penetration into the core might change the core size and also other parameters derived based on the size of the core, such as *N*_agg_.

Our obtained results from the FCC model for P407 are in line with the SANS study on P403 showing a higher core radius upon addition of EAN. Since high scattering noise prevented reliable model fitting for the water-containing sample in our study, however, we cannot confidently rule out the trend reported by Chen et al.

Figure S1a and b show the dynamic moduli of P100EAN in the temperature ramp tests with strain amplitude of 0.1 and 10%, respectively. At a strain amplitude of 0.1%, the stress curves are sinusoidal in the gel state, while the noise to signal ratio is high in the sol state, suggesting that reported storage and loss moduli by the instrument become unreliable in this state. At a strain amplitude of 10% on the other hand, the stress curves are close to a sine curve in the sol state, whereas the stress curves in the gel state show that the material undergoes nonlinear deformation. It should be noted that although higher strain amplitude is selected for rheological measurements in the sol state, *G*′ is still scattered in this regime due to low viscosity. Therefore, the reported moduli of strain amplitude of 0.1% for the gel state and 10% for the sol state is combined to produce the corrected temperature ramp plots in Figure 2 and Figure S1c.

**Figure 2:**
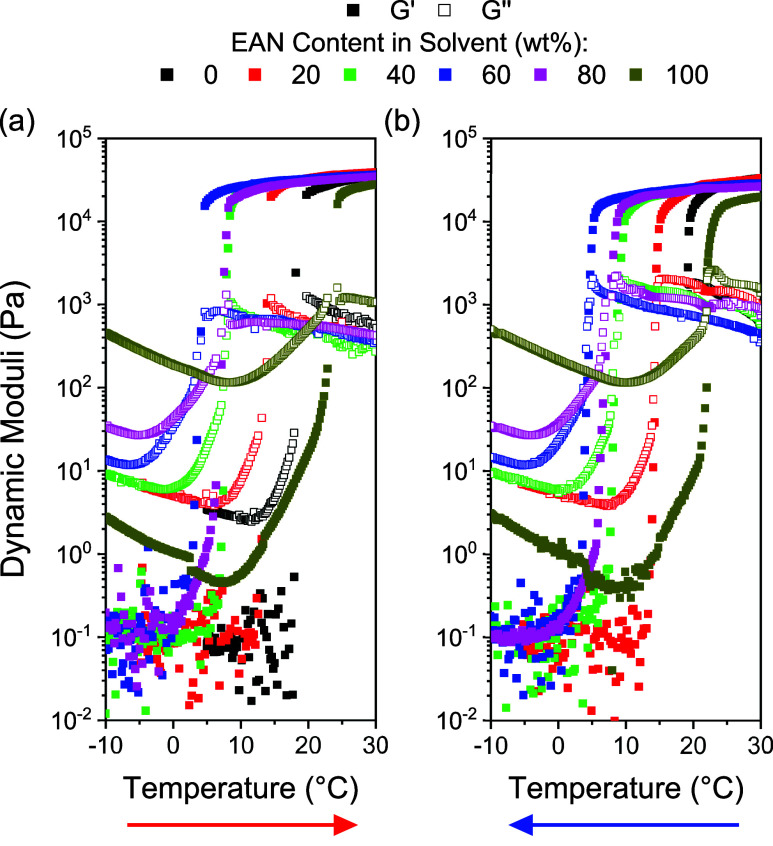
Dynamic moduli of samples with different EAN content vs temperature in (a) heating and (b) cooling experiments with temperature ramp of 2 °C/min and frequency of 40 rad/s. For each sample, the data for the sol state is plotted from measurements with strain amplitude of 10% and the data for the gel state is plotted from measurements with strain amplitude of 0.1%.

Different rheological measures have also been considered for determining the sol–gel transition; e.g., a few orders of magnitude sharp increase of viscosity^60,61^ or elastic modulus.^45^ In the case of aqueous P407 systems, the flow curve has been studied at different temperatures, in which the lowest temperature with signs of non-Newtonian behavior, particularly yield stress, is considered as the gelation temperature.^62^ Poloxamer systems have Newtonian-like behavior in the sol state and an elastoviscoplastic behavior in the gel state which can be fitted with Herschel–Bulkley model.^62,63^ Considering gels having a solid-like behavior (i.e., *G*′ > *G*″) and sols having a liquid-like behavior (i.e., *G*′ < *G*″), the crossover of *G*′ and *G*″ has been traditionally used to determine the sol–gel transition point.^64–66^ In this work, the frequency and the strain amplitude are chosen in a way that the crossover of moduli is observed for determining the sol–gel transition temperature, *T*_gel_. Figure S2 presents the flow curves of samples in the sol state, demonstrating their Newtonian behavior with constant viscosity across shear rates.

*G*′ shows an approximately 5 orders of magnitude increase during gelation. The sol–gel transition is reversible, as confirmed by several cycles of heating and cooling. Looking closely at the *T*_gel_ in samples with different solvent compositions shows that P0EAN and P100EAN samples have a *T*_gel_ of approximately 18 and 23 °C in the heating cycle, respectively. However, thermal hysteresis plays a role here, and *T*_gel_ in the cooling cycle is higher than that of the heating cycle by up to about 1 °C in all samples except P100EAN, which is the other way around.

The results show that *T*_gel_ does not change linearly by increasing the EAN content in the solvent. Interestingly, a negative deviation from the mixing rule is observed with a minimum at 60 wt % EAN, exhibiting a *T*_gel_ of approximately 4 °C. It is well-known that the sol–gel transition in this system is attributed to the disorder–order transition from a micellar solution to a highly ordered mesophase with FCC structure. This process is analogous to increasing the concentration of the surfactant at a fixed temperature. Gelation of P407 solution is believed to result from entropically driven micellization and spatial packing constraints at high concentrations, where micelles interact to form structured phases while remaining intact.^16^ Similar to water-based LLC systems, López-Barrón et al. attributed gelation in IL-based LLC systems to the weakened hydrogen bonding upon heating.^45^ Dehydration of PPO blocks has also been suggested as the driving force of the gelation in aqueous poloxamer solutions.^67^ It has also been speculated that the gelation can be entropically driven by ordering of water molecules taking place near to the hydrophobic core.^68^

It is suggested that micelles behave like hard spheres in high concentrations. Consequently, when their volume fraction reaches about 0.53, micelles crystallize into a “hard-sphere cubic crystal”, causing gelation and structural ordering evidenced by a sharp increase in viscosity.^69^ It should be noted that other transitions at temperatures higher than sol–gel transition have also been observed. Liu and Li observed a hard gel-soft gel transition in the range of 70–90 °C for P407 in water gels at different concentrations. They suggested that shell becomes desolvated at higher temperatures and part of PEO merges into the PPO core resulting in larger cores, smaller shells, and consequently lower degree of overlapping of micelles coronas leading to reduced entanglement density of PEO chains and a hard–soft gel transition.^48^ This order–order transition has been observed in other studies, too.^70,71^ The PEO shell dehydration at high temperatures can lead to the breakup of the gel lattice and result in a thermally reversible transition from a gel to a sol state.^72^

Starting from low temperatures, PPO blocks are soluble in water and hydrophilic, enabling surfactants being present as unimers in the solution without any sign of micelle. Upon heating, PPO blocks become desolvated and more hydrophobic to an extent that at *T*_mic_, micelles with hydrophobic PPO cores are formed.^63^ Looking at the heat flow of P100EAN in Figure 3a, an endothermic peak is observed in the range of 6–20 °C followed by maintaining the baseline at higher temperatures. Such broad endothermic peak has been reported for aqueous solutions of different poloxamers including P407 at moderate concentrations, where the onset of the peak determines *T*_mic_.^48^ Presence of this peak in the corresponding cooling cycle shows demicellization, proving reversibility of micellization.

**Figure 3:**
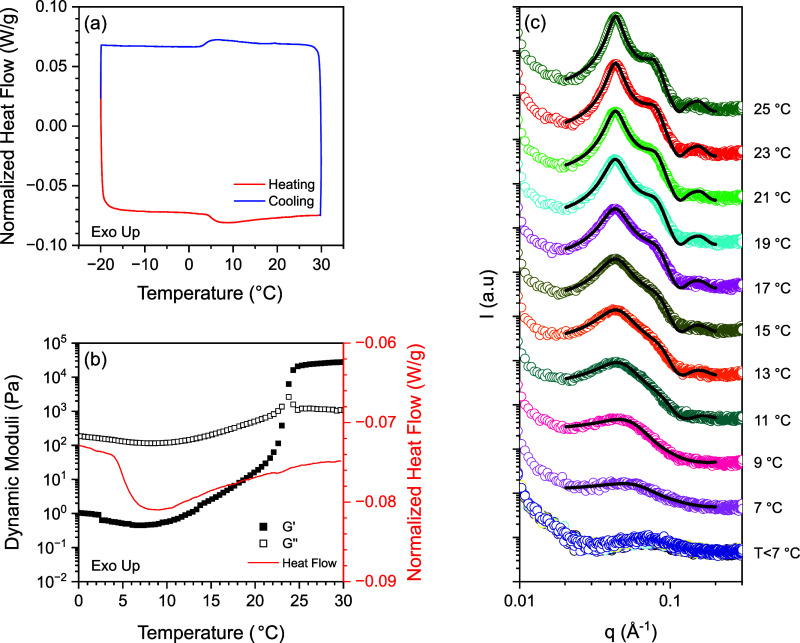
Effect of temperature on the structure and flow behavior of P100EAN: (a) thermal transitions of the mesophase in heating and cooling cycle obtained from DSC, (b) rheological behavior of the mesophase upon increasing the temperature compared with DSC thermograms, and (c) scattering of P100EAN at different temperatures upon heating obtained from SAXS. Black lines are fittings to core–shell form factor and hardsphere structure factor using eqs S1–S5.

Since formation of thermodynamically stable micelles is spontaneous (Δ*G* ≤ 0), endothermic enthalpy change (Δ*H* ≥ 0) makes it enthalpically unfavorable. Thus, the entropy change, Δ*S*, should be positive, making micellization process entropy-driven.^16^ This might seem counterintuitive when comparing unimers and micelles. This discrepancy could be explained based on the hydrophobic effect in traditional micelle formation theory. Dispersion of hydrophobic moieties of surfactants in water disrupts the hydrogen-bonding network of water, causing water molecules to become more ordered and reducing their entropy. This “cage” of structured water molecules around the hydrophobic moieties is known as the “iceberg” model.^73^ Upon aggregation of hydrophobic chains to form micelles, the majority of water network is restored due to lower surface area formed around aggregates, freeing water molecules and increasing their entropy. This increase in water entropy offsets the entropy loss from the ordered hydrophobic cores within micelles, making micellization a spontaneous, entropy-driven process. The endothermic nature of micellization is due to hydrophobic interactions and required energy to break hydrogen bonds between water molecules in the “cage”.^68^

Looking more closely at the thermal transitions and dynamic moduli at different temperatures in Figure 3b shows a small endothermic peak near to the crossover of storage and loss moduli. Similar endothermic transitions with less significant enthalpy compared to micellization have been attributed to gelation in the literature.^74,75^ This small enthalpic change is due to intermicellar interactions. Alexandridis et al.^76^ reported steric repulsion of solvated PEO shells as the reason for close-packing of micelles into cubic arrays. LLC modulus has also been successfully modeled by considering van der Waals (VdW) forces in cubic arrangement of spherical micelles.^77^ Assuming the sol–gel transition as a reversible equilibrium phase transition, Δ*G*_gel_ = 0 and changes in entropy of gelation can be obtained by Δ*H*_gel_ = *T*Δ*S*_gel_. Since the Δ*H*_gel_ is positive, as evident by the DSC results, Δ*S*_gel_ should be positive, which seems to be counterintuitive considering gelation as a disorder–order transition. This behavior could be explained based on the increased entropy of water molecules in the confinement of hydrophobic domains, being PPO core of micelles in this system that induces nanoconfinement on solvent molecules upon LLC formation. As a result, the process is driven by this gain in entropy, despite forming a more structured gel network.^68,78,79^

Looking at the scattering patterns of P100EAN starting from temperatures below observed *T*_mic_ in DSC (<7 °C), no scattering other than the background and scattering from polymer chains (unimers) in the form of a broad peak is observed at *q*-values above 0.02 Å^–1^ (see Figure 3c). Determining *T*_mic_ by SAXS is done by determining the first temperature at which a mid-*q* signal becomes evident. Starting at *T*_mic_, the structure factor peak around *q* value of 0.04 Å^–1^ starts to build up in the scattering profile, as can be seen in Figure S3. Therefore, the intersection of two linear trends can be used to determine *T*_mic_. Distinctive peaks with Bragg peak ratios of $\sqrt{3}:\sqrt{4}:\sqrt{8}:\sqrt{11}$ are observed at temperatures above 25 °C, confirming LLC formation with FCC structure. It should be noted that SAXS experiments have been performed with temperature increment of 2 °C. Thus, *T*_mic_ and *T*_gel_ are determined as the average between two temperatures in which the SAXS experiments show a clear transition with accuracy of ±1 °C. For instance, *T*_mic_ of P100EAN is determined to be 6 ± 1 °C and *T*_gel_ for this sample is 24 ± 1 °C. Spherical core–shell form factor with hard sphere structure factor is used to fit the SAXS data at different temperatures and obtain structural information such as *R*_core_; shell thickness, *L*_shell_; micelle radius, *R*_mic_ = *R*_core_ + *L*_shell_; aggregation number, *N*_agg_; volume fraction of micelles in the system, φ_mic_; and volume fraction of solvent in shell, φ_s,s_. Similar plots for samples with different EAN contents have been shown in Figures S4–S8.

Figure 4 shows the obtained *T*_mic_ and *T*_gel_ from methods used in this study for samples with different EAN contents in both heating and cooling cycles with an onset showing changes in *T*_gel_ – *T*_mic_ against EAN content in the solvent. This plot shows that *T*_mic_ and *T*_gel_ have the same trend with a minimum observed for P60EAN. Similar behavior has been observed upon adding EAN to the aqueous solvent in self-assembly of other poloxamers. He and Alexandridis^38^ reported that adding ∼1.75 M EAN to an aqueous P403 solution has similar micellization-promoting effect as raising temperature from 20 to 24 °C, which is attributed to the decrease of CMC in both cases. In other words, addition of EAN to the same concentration of surfactant lowers the *T*_mic_ from 24 to 20 °C. EAN effectively “salts out” the block copolymer, similar to the effect of adding inorganic salt or increasing temperature.^38^ Madhusudhana Reddy and Venkatesu^41^ found that IL additives universally lowered the *T*_mic_ of P338 in water, but the magnitude depended on the IL anion and its concentration.

**Figure 4:**
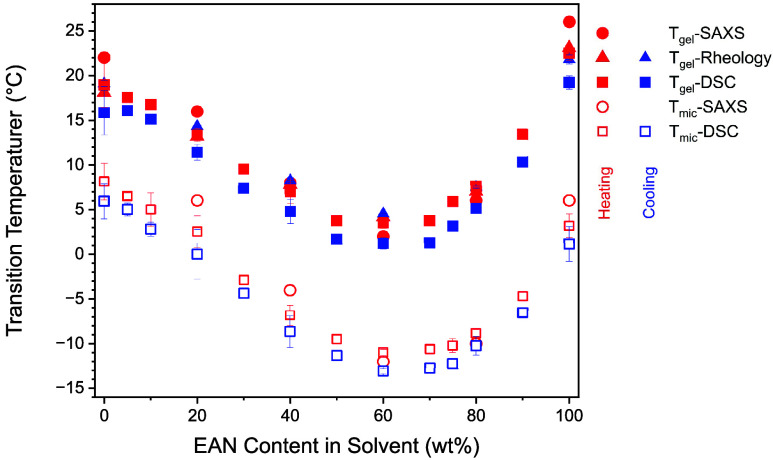
*T*_mic_ and *T*_gel_ were determined by different methods for samples with different EAN contents.

The key to inducing micellization by changing temperature is the temperature dependence of PEO–PEO, PPO–PPO, and PEO–PPO interactions. Increasing the temperature weakens the PEO–PPO repulsion. At *T*_mic_, hydrophobic attraction of PPO–PPO dominates PEO–PEO repulsion, resulting in micelle formation.^67^ A crucial property of a solvent that supports effective amphiphile self-assembly is its high cohesive energy density, which is usually observed in liquids capable of forming hydrogen bonds.^30^ Higher cohesive energy density results in maintaining the hydrogen bonding and solvent structure around the hydrophobic part of the surfactant and increases the solvophobicity of PPO blocks. Thus, amplified solvophobicity of PPO in a solvent increases the attraction between PPO blocks, implying that the PPO–PPO attraction-dominant regime is induced at lower temperatures. Consequently, the micellization temperature decreases with enhanced cohesive energy density of solvent.

Protic ILs such as EAN have protons available on the cationic part as a hydrogen bond donor, which enables EAN to form a three-dimensional hydrogen-bonded network similar to water.^30^ However, formation of liquid intermediate-range order with segregated polar and nonpolar domains is observed in pure EAN due to the presence of the ethyl moiety on the cation in the EAN structure.^80^ Mixtures of water and EAN contain hydrogen bonding and show negative excess molar volume at nearly all compositions due to the incorporation of alkane molecules into the gaps within the hydrogen-bonding network of water.^42^ A minimum excess molar volume has been observed based on the Redlich–Kister equation at around 60–80 wt % EAN in water, which indicates the strongest hydrogen bonding strength at this composition.^81^ In the water-rich region (below 60 wt %), ion pairs begin to dissociate, resulting in the presence of water-solvated cations and anions, along with free water molecules that are not involved in hydrogen bonding with EAN. In the EAN-rich region, however, ion pairs and clusters are observed.^82^ The variation in hydrogen bonding of EAN/water mixtures explains the minimum *T*_mic_ observed at 60:40 wt %. Between pure water and EAN, considering the stronger hydrogen bonding in water compared to EAN, lower *T*_mic_ in P100EAN compared to P0EAN could be due to the presence of nearly 1.4 wt % residual water and other impurities in the EAN used as received.

As the EAN fraction increases in the solvent, *T*_gel_ – *T*_mic_ increases, too. Investigating the structural parameters obtained by core–shell model is essential to understand the process in which micelles undergo to finally form the LLC structure. Since the *T*_mic_ and *T*_gel_ are not the same for different samples, comparing the structural parameters at a fixed temperature is not helpful. Thus, as proposed by Mortensen and Brown,^83^ we use the relative temperature, *T*_r_, defined as *T*_r_ = *T* – *T*_mic_ to eliminate the effect of solvent. Studied structural parameters of the micelles are plotted at different *T*_r_ in Figure 5 and the corresponding data are tabulated in Tables S2–S6. *R*_core_ shows a sharp increase in the first 5 °C after micellization and reaches a plateau after which it has a slight slope. From the trend of changes in *N*_agg_, we conclude that unimers continue to join the micelle core until *T*_r_ ≈ 5 °C as the *N*_agg_ increases from ∼10 to ∼35 in this temperature range while the rate of this process slows down significantly afterward with *N*_agg_ changing from ∼35 to ∼40. A more detailed analysis is presented in Figure 5e.

**Figure 5:**
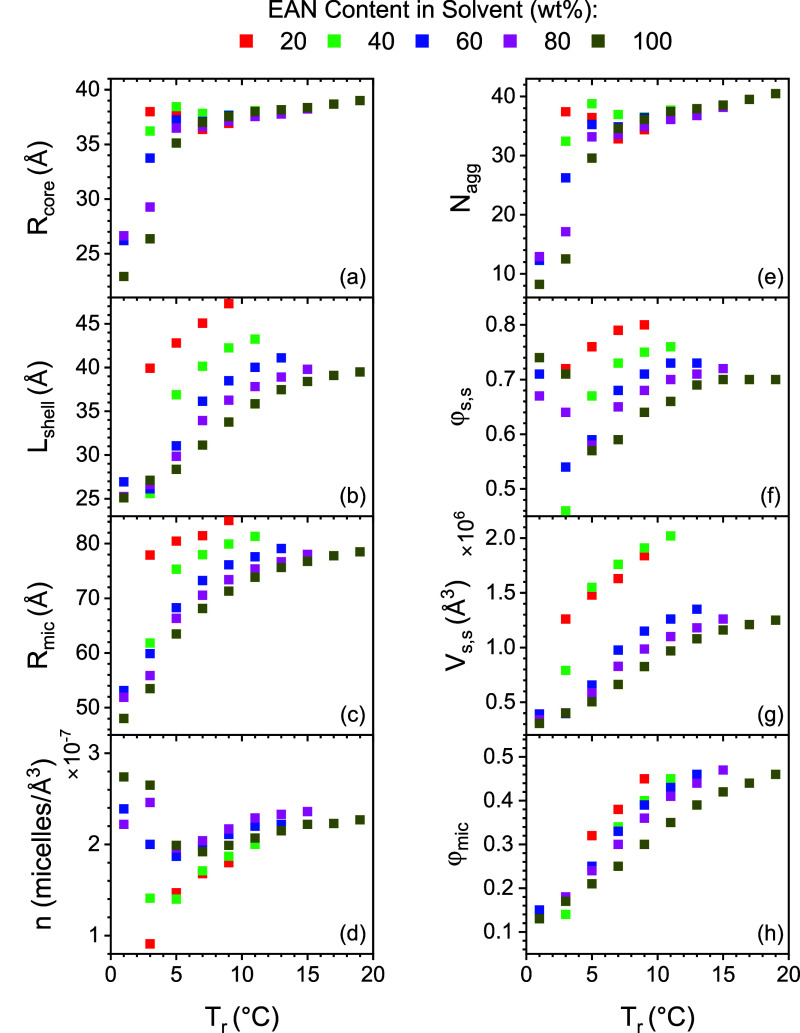
Changes in (a) core radius, *R*_core_, (b) shell thickness, *L*_shell_, (c) micelle radius, *R*_mic_, (d) number of micelles per unit volume, (e) aggregation number, *N*_agg_, (f) volume fraction of solvent in shell, φ_s,s_, (g) volume of solvent in the shell, and (h) volume fraction of micelles in the system, φ_mic_, vs *T*_r_ upon heating from *T*_mic_ to *T*_gel_. Values are obtained by fitting a core–shell form factor with hard sphere structure factor on the SAXS data.

Changing the solvent composition from water-rich to EAN-rich does not have a significant effect on *R*_core_. This observation is in accordance with reports in the literature and is mainly due to the similar solubility of PPO in EAN and water.^39,59^ For samples with different solvent content, *R*_core_ and *N*_agg_ before gelation vary in the range of 36–39 Å and 31–41 Å, respectively. The reported value for *R*_core_ is in the same range of sphere radius obtained by FCC model with paracrystal distortion. Thus, we believe that the hard sphere detected by the model is the core in the core–shell spherical micelles, further confirming that core is mainly composed of dry PPO. The empirical scaling relation of *R*_core_ ∝ *T*_r_^0.2^ has been reported for different poloxamers with the same PPO block size of 40 units and different concentrations.^84^ Based on the negligible effect of solvent on core size, it may be expected that our results follow the same trend. Fitting the data with a power function gives the empirical relation of *R*_core_ ∝ *T*_r_^0.116^ for P407 with PPO block size of 65 (see Figure S9). Further investigation is needed to confirm whether this relation depends on the concentration or not.

The shell of the micelles is believed to be made of solvated PEO blocks. Using the *N*_agg_ calculated based on the core size, dry shell volume, *V*_dry_, made of only PEO blocks can be calculated, eq 6, to obtain the volume fraction of solvent, eq 7, in the shell using the following equations:^47^

6 $$V_{\mathrm{dry}}=\frac{N_{\mathrm{agg}}}{N_{A}}\frac{M_{w,\mathrm{PEO}}}{\rho_{\mathrm{PEO}}}$$

7 $$\varphi_{s,s}=\frac{{V_{\mathrm{shell}}-V}_{\mathrm{dry}}}{V_{\mathrm{shell}}}$$

where ρ_PEO_ is the bulk density of PEO, *M*_w,PEO_ is the molecular weight of PEO block in P407, and *V*_shell_ is the volume of the shell. Adding more EAN to the solvent reduces *L*_shell_ from ∼47 Å in P20EAN to ∼39 Å at a temperature just below gelation. Although the *N*_agg_ is higher in samples with higher EAN content, φ_s,s_ decreases from ∼0.8 to ∼0.7, which shows the solvation of the shell is not a direct function of the number of PEO chains. This observation clearly shows that compared to EAN, water is a better solvent for PEO as evident from the smaller radius of gyration of PEO in EAN (8.1 nm) than that of water (9.6 nm).^85^ The results are in agreement with the reports in the literature. Chen et al. reported approximately 33% reduction in shell thickness in P403 micelles (5 wt %) by replacing water with EAN, while the core radius remained almost the same.^39^ Zhang et al. showed that increasing the concentration of EAN in the aqueous solvent up to 2 M decreased the shell thickness by 5% and 8% at 20 and 40 °C, respectively.^59^ The general trend in φ_s,s_ is the same as *L*_shell_. However, due to the initial sharp increase in the aggregation number, the φ_s,s_ decreases at first and resumes the increasing trend after *T*_r_ ≈ 5 °C. The solvent volume in the shell, *V*_s,s_ = *V*_shell_ – *V*_dry_ (Figure 5g), changes slightly in the *T*_r_ range of 0–5 °C, which shows its weaker dependence on the changes in *N*_agg_.

Micelle radius can be calculated by adding up *R*_core_ and *L*_shell_. *R*_mic_ increases for all samples upon increasing *T*_r_ until reaching *T*_gel_. Since *R*_core_ is almost the same for all samples up to 1 °C before gelation, *R*_mic_ at this *T*_r_ follows the same general trend as *L*_shell_. Therefore, systems with higher EAN content have smaller micelles varying from ∼86 Å for P20EAN to ∼78 Å for P100EAN.

Figure 5h shows the evolution of φ_mic_ by increasing *T*_r_ for different solvents. φ_mic_ increases almost linearly with *T*_r_ for all samples. However, the slope and intercept of a hypothetical linear fitting vary for different solvents. The final φ_mic_ at the last point before gelation varies between 0.46 and 0.47, which suggests that gelation happens at a specific φ_mic_. Further extrapolations determine the critical φ_mic_ at the gelation point, which is evaluated in Figure 6 and will be discussed later. It has been suggested that the number of micelles grow by increasing the temperature after micellization.^48^ To validate this hypothesis in our work, micelle number density, *n̂*, which is equal to the number of micelles, *n*, per unit of total volume, *V*, is calculated using eq 8 and presented in Figure 5d:

8 $$\mathrm{n̂}=n/V=\frac{\varphi_{\mathrm{mic}}}{4/3\pi R_{\mathrm{mic}}^{3}}$$

**Figure 6:**
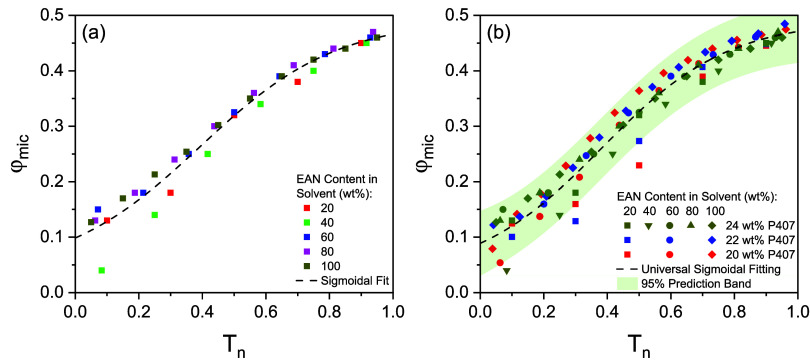
Changes in φ_mic_ against the normalized temperature, *T*_n_, for samples with different EAN/water weight fractions at (a) 24 wt % P407 and (b) different P407 concentrations. Dashed lines show the sigmoidal fitting on the superimposed data.

The results show that *n̂* initially decreases before reaching *T*_*r*_ ≈ 5 °C, after which it begins to increase. Considering the monotonic increasing trend in *R*_mic_, *N*_agg_ and φ_mic_, we propose the following mechanism to explain the structural changes of micelles upon heating from *T*_mic_ to *T*_gel_. The observed decrease in the number density of micelles at *T*_r_ ≈ 5 °C suggests that small micelles coalesce, leading to growth in micelle size which can be observed by the increasing trend in both *R*_mic_ and *N*_agg_. However, since φ_mic_ is not constant during this period, it can be concluded that free unimers from the solution are also incorporating into micelles, further contributing to the rise in micelle volume fraction. We hypothesize this coalescence of micelles can be explained through micelle fusion which has been reported for polybutadiene-PEO micelles in the 1-butyl-3-methylimidazolium tetrafluoroborate IL, and is attributed to reduced solubility of PEO in the IL and reduced osmotic repulsion between the PEO chains, lowering the steric barrier to micelle fusion.^86^ The likelihood of fusion increases at high micelle concentrations where micelles collide more frequently. Initial high micelle count in our case thus is hypothesized to enhance the fusion. However, this hypothesis requires further experimental proof such as cryo-transmission electron microscopy (cryo-TEM) studies.^86^

It cannot be concluded that the fusion process ceases above *T*_r_ ≈ 5 °C. However, it is clear that formation of new micelles by free unimers contributes to the increased micelle number density at higher temperatures. Therefore, fusion appears to dominate at temperatures below *T*_r_ ≈ 5 °C, while micelle growth becomes dominant at higher temperatures. When the temperature approaches *T*_gel_, micellization rate diminishes as the micelle count tends to level off.

The critical micelle volume fraction needed for LLC structure formation is believed to be independent of polymer concentration. As shown in Figure 5h, the solvent effect on changes in φ_mic_ appears in the slope of φ_mic_ vs *T*_r_. Since *T*_gel_ – *T*_mic_ increases at higher EAN contents in the solvent, we define the normalized temperature, *T*_n_, as in $T_{n}=\frac{T-T_{\mathrm{mic}}}{T_{\mathrm{gel}}-T_{\mathrm{mic}}}$ to remove the solvent effect. Figure 6a plots the changes in φ_mic_ against *T*_n_ for samples with different EAN contents in the solvent. All data points are superimposed with a sigmoidal correlation in the generic form of a Boltzman sigmoid function, as in eq 9:

9 $$y=\frac{y_{i}-y_{f}}{1+\exp\left(\frac{x-x_{50}}{dx}\right)}+y_{f}$$

where *y*_i_ and *y*_f_ are the initial and final values at which the trend plateaus, *x*_50_ is the center of the curve at which *y* is halfway between *y*_i_ and *y*_f_, and *dx* is the slope factor. Fitting data of 24 wt % P407 shows the sigmoidal function of ${\varphi_{\mathrm{mic}}}^{24\mathrm{wt}\%P407}=\frac{0.035-0.492}{1+\exp\left(\frac{T_{n}-0.393}{0.216}\right)}+0.492,\left(R^{2}=0.97\right)$ .

To check the effect of surfactant concentration on this trend, Figure 6b plots the φ_mic_ against *T*_n_ with different EAN contents in the solvent at 20, 22, and 24 wt % P407. This plot reveals that the same trend is present for different surfactant concentrations, which can be fitted to obtain a universal trend in eq 10 (*R* ^2^ = 0.96) to estimate the volume fraction of micelles only by knowing the *T*_mic_ and *T*_gel_.

10 $$\varphi_{\mathrm{mic}}=0.491-\frac{0.463}{1+\exp\left(\frac{T_{n}-0.381}{0.201}\right)}$$

The upper bound of 0.491, which indicates the critical volume fraction of micelles for FCC structure formation, agrees with the literature. It has been reported that hard spheres form FCC crystalline ordering above a volume fraction of 0.498 to minimize the total energy of the system by maximization of the entropy.^87^ A similar concept has been used to explain FCC formation of block copolymer solutions above a critical volume fraction of 0.53, which implies soft colloidal spheres have the same behavior as hard spheres.^69,88–90^ Our results show that the entropy-driven crystallization of hard spheres also applies to soft micelles of P407.

Figures S11–12 and Tables S7–12 show the scattering profile and the obtained values from fittings of samples with 20 and 22 wt % P407, which are summarized and compared with 24 wt % P407 in Figure S13. Observed trends, including two distinct regimes of changes in the number density of micelles, are persistent at different surfactant concentrations. At all solvent compositions, increasing the surfactant concentration increases *N*_agg_, resulting in slightly larger *R*_core_. However, micelles are slightly smaller at higher surfactant concentrations, which indicates a smaller *L*_shell_ that arises from lower solvent incorporation into the shell. This observation suggests that the number of solvent molecules per PEO chain decreases as the number of PEO chains increases. However, this hypothesis should be further investigated in a more systematic manner. This discrepancy between the trends of *R*_mic_ and *N*_agg_ is offset by the higher number density of the micelles at higher surfactant concentrations.

## Conclusion

In this study, we have systematically investigated the self-assembly and micellization behavior of Poloxamer 407 in mixtures of water and EAN, revealing key insights into how solvent composition and temperature modulate micellar architecture and mesophase formation. Our results reveal that solvent composition notably influences the critical micellization temperature, *T*_mic_, sol–gel transition temperature, *T*_gel_, and micellar structure with a nonmonotonic trend observed. Both *T*_mic_ and *T*_gel_ show minima at 60 wt % EAN, reflecting optimal hydrogen bonding and solvophobic interactions at this composition.

The detailed micellization mechanism shows a multistep process with distinct temperature-dependent structural transitions from unimers to micelles to the FCC packing of micelles. While the aggregation number, micelle radius, and micelle volume fraction increase steadily in the micellar regime, a decrease in micelle number density is dominant at early stages of micellization, reaching a minimum followed by an increase in number density. Although the whole micellization mechanism is consistent within different solvent compositions, increasing the EAN content reduces solvent incorporation in the shell.

Transitioning from micelles to self-assembled structures, the critical micelle volume fraction for FCC ordering remains ∼0.49 across all solvent compositions, suggesting that gelation and FCC lattice formation are governed primarily by spatial packing constraints and entropic bonding rather than specific solvent–polymer interactions. Since the micelles in EAN-rich solvents are smaller and more compact due to lower solvent incorporation in the shell, the temperature gap between micellization and gelation increases almost linearly by increasing the EAN content in the solvent. This result suggests higher temperatures are required for smaller micelles to reach the universal critical volume fraction for packing into the ordered FCC structure. Notably, we have defined a normalized temperature. The change in volume fraction of micelles versus normalized temperature introduced here shows a superposition across all solvent systems at different surfactant concentrations, collapsing into a single sigmoidal trend. We hypothesize that the trends observed here apply to different poloxamers; however, further investigations are needed to confirm.

## Supplementary Material



## Data Availability

The experimental data are available from the authors upon reasonable request.

## Notes

Scattering models; Rheological temperature ramp test protocol; Sol state flow curve; Intensity of the structure factor peak of 24 wt % samples at different temperatures; Combined DSC, rheology, and SAXS data for 24 wt % samples; Core radius fitting; Changes in structure of micelles vs *T*_n_; SAXS data and fitting results for samples with 20 and 22 wt % P407; Micellization mechanism vs *T*_n_; Sample matrix; Core-shell analysis results for all samples (PDF)
