# Supplementary material for: From Solvent-Mediated Micellization to Packing in a Face-Centered Cubic Structure of Poloxamers
Source: Macromolecules. 2026 Jun 9;59(12):7131–43. doi: 10.1021/acs.macromol.6c00316 (PMC13296491; doi:10.1021/acs.macromol.6c00316)
Supplement: Supplementary file 1 [file ma6c00316_si_001.pdf]

Supporting Information

From Solvent-Mediated Micellization to Packing in a Face-Centered Cubic Structure of Poloxamers

Seyed Mostafa Tabatabaei[1], Javen Weston[2], John Klier[1], Reza Foudazi[1*]

[1] School of Sustainable Chemical, Biological and Materials Engineering, The University of Oklahoma, Norman, OK 73019, USA

[2]Chemical Engineering Department, University of Tulsa, Tulsa, OK, 74104, USA

Scattering Models

The scattering intensity, I, of a monodisperse system obtained by SAXS can be written as a function of the wave vector, q, as follows:[48]

$$I(q) = \Delta\rho^2 N P(q) S(q) \quad \text{(S1)}$$

In which $\Delta\rho^2$ is the contrast factor with ρ as the scattering length density (SLD), N is the number density of particles, $P(q)$ is the form factor indicating the shape and structure of the micelles, and $S(q)$ is the structure factor indicating the interactions between micelles. The spherical core-shell form factor and hard sphere structure factor are fitted onto the scattering data using SasView according to eq. (2-5).[49,50] It is worth mentioning the signal from P0EAN was weak, which made fittings of its scattering data unsuccessful. The form factor for a spherical core-shell sphere, $P_{cs}(q)$, is given by:[51]

$$P_{cs}(q) = \frac{\text{scale}}{V} F^2(q) + \text{background} \quad \text{(S2)}$$

---

* Corresponding author. Email: rfoudazi@ou.edu.

where:

$$F(q) = 3\left[V_c(\rho_c - \rho_s)\frac{\sin(qR_c)-qR_c\cos(qR_c)}{(qR_c)^3} + V_{mic}(\rho_{sh} - \rho_{\text{solv}})\frac{\sin(qR_{mic})-qR_{mic}\cos(qR_{mic})}{(qR_{mic})^3}\right] \quad \text{(S3)}$$

In which V is volume, R is radius, subscript c denotes the core, subscript mic denotes the whole micelle (core+shell), and subscript $solv$ denotes the solvent. Hard sphere structure factor, $S_{hs}(q)$, is given by:[52]

$$S_{hs}(q) = 1 + 4\pi n\int_0^\infty [g(r) - 1]\frac{\sin(qr)}{qr}r^2\,dr \quad \text{(S4)}$$

In which n is the particle number density, and $g(r)$ is the radial distribution function calculated by Percus-Yevick closure relationship[53] where the interparticle potential, $U(r)$, is assumed to be:

$$U(r) = \begin{cases} \infty & r < 2R \\ 0 & r \geq 2R \end{cases} \quad \text{(S5)}$$

where r shows the distance from the center of a sphere with radius of R.

To obtain structural information from LLC structures at higher temperatures, the SAXS data were fitted by SasView using FCC lattice model with paracrystalline distortion, eq. (6)-(11), which assumes monodispersed spherical particles arranged in an infinitely extended cubic lattice, incorporating isotropic paracrystalline distortions. This model computes the resulting scattering intensity, $I_{FCC}(q)$, as: [54]

$$I_{FCC}(q) = \frac{\text{scale}}{V_p}V_{\text{lattice}}P_{ss}(q)Z(q) + \text{background} \quad \text{(S6)}$$

where V_{lattice} represents the volume occupied by the spheres within the crystal structure and V_p denotes the volume of an individual primary particle. $P_{ss}(q)$ is a simple spherical form factor given by:[51]

$$P_{ss}(q) = \left[3\left(\frac{\sin(qR) - qr\cos(qR)}{(qR)^3}\right)\right]^2 \tag{S7}$$

In which R is the radius of the sphere. $Z(q)$ is the paracrystalline structure facture which can be calculated using the following equation:[55]

$$Z(\vec{q}) = \prod_{k=1}^{3} Z_k(\vec{q}) \tag{S8}$$

where:

$$Z_1(q, \theta, \phi) = \frac{1 - e^{-q^2\Delta a^2}}{1 - 2e^{-\frac{1}{2}q^2\Delta a^2}\cos\left[\frac{qD}{\sqrt{2}}(\sin\theta\cos\phi + \sin\theta\sin\phi + \cos\theta)\right] + e^{-q^2\Delta a^2}} \tag{S9}$$

$$Z_2(q, \theta, \phi) = \frac{1 - e^{-q^2\Delta a^2}}{1 - 2e^{-\frac{1}{2}q^2\Delta a^2}\cos\left[\frac{qD}{\sqrt{2}}(-\sin\theta\cos\phi - \sin\theta\sin\phi + \cos\theta)\right] + e^{-q^2\Delta a^2}} \tag{S10}$$

$$Z_3(q, \theta, \phi) = \frac{1 - e^{-q^2\Delta a^2}}{1 - 2e^{-\frac{1}{2}q^2\Delta a^2}\cos\left[\frac{qD}{\sqrt{2}}(-\sin\theta\cos\phi + \sin\theta\sin\phi - \cos\theta)\right] + e^{-q^2\Delta a^2}} \tag{S11}$$

In these equations, a is the unit cell lattice parameter and D is the nearest-neighbor distance.

In all fittings, the SLD of core is fixed assuming a dry core made of PPO with SLD of 9.48×10^{-6} Å^{-2}.[38] Considering water and EAN having SLD of 9.45×10^{-6} Å^{-2} and

11.08×10^{-6} Å^{-2},[38] respectively; the SLD of solvent mixture is calculated and fixed in models using the following equation where φ is the volume fraction.

$$\rho_{\mathrm{solv}} = \varphi_{EAN} \times \rho_{EAN} + (1 - \varphi_{EAN}) \times \rho_{water} \qquad \text{(S12)}$$

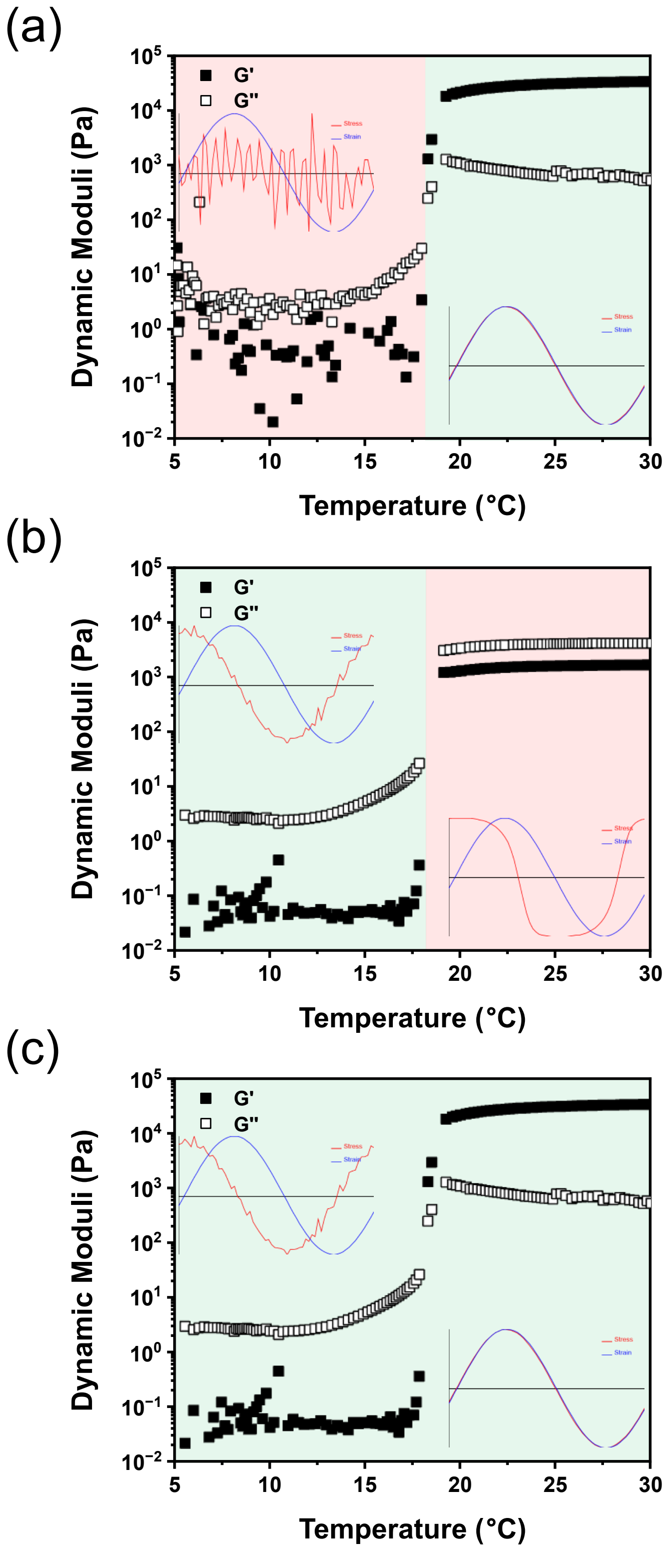

Figure S1. Changes in dynamic moduli vs temperature at frequency of 40 rad/s and strain amplitude of (a) 0.1%, (b) 10%, and (c) combined data in strain amplitude of 0.1% for the gel state and 10% for the sol state.

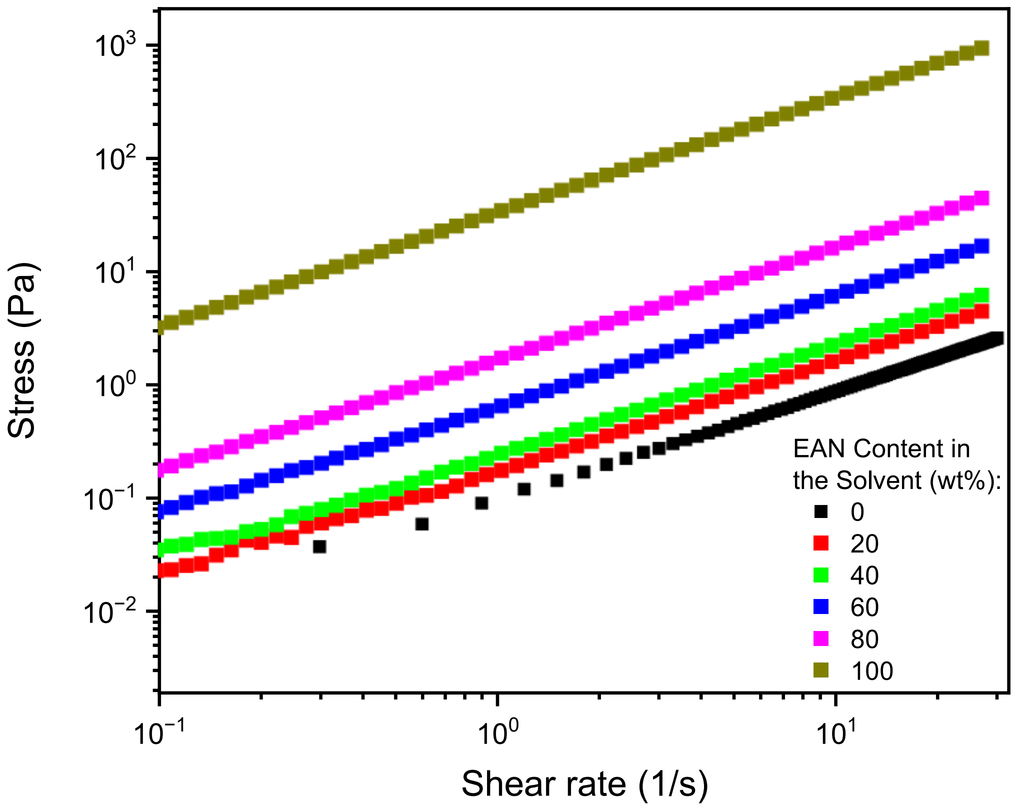

Figure S2. Flow curve of samples with different EAN contents at −20 ℃, except P0EAN which was tested at 5 ℃ to avoid freezing the sample.

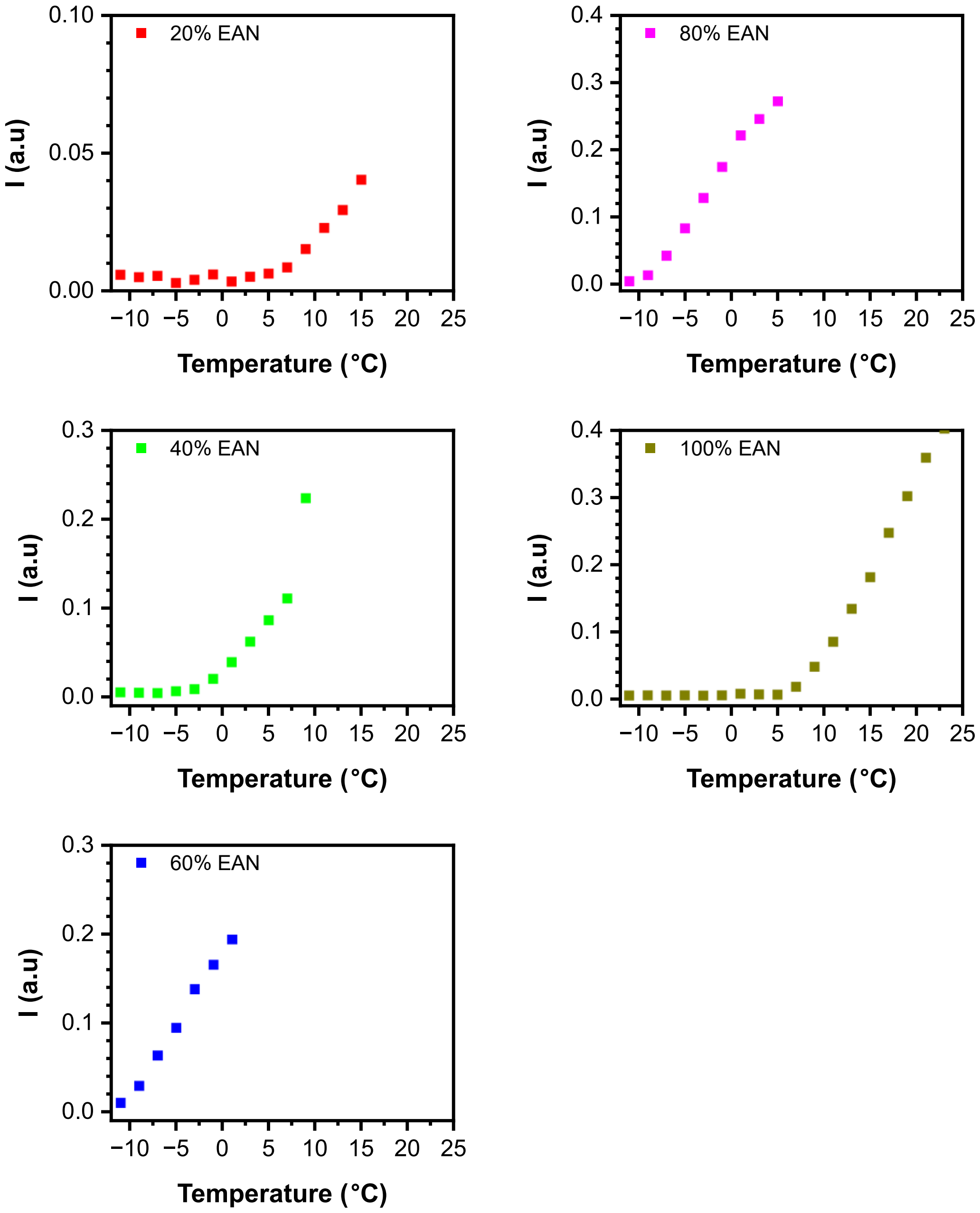

Figure S3. Intensity at the q-value of structure factor peak for different samples to be used for determination of T_{mic}

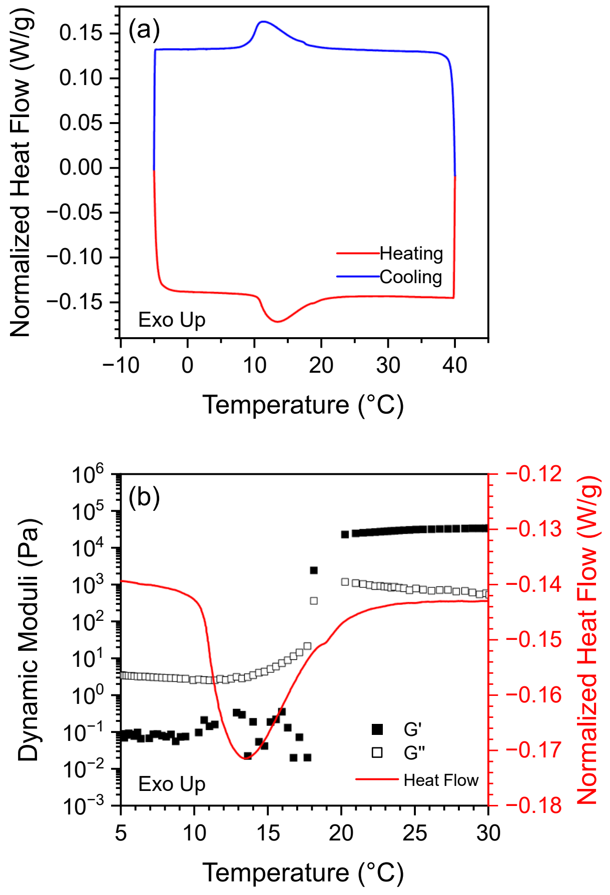

Figure S4. Effect of temperature on the structure and flow behavior of P0EAN: (a) thermal transitions of the mesophase in heating and cooling cycle obtained from DSC, (b) rheological behavior of the mesophase upon increasing the temperature compared with DSC thermograms.

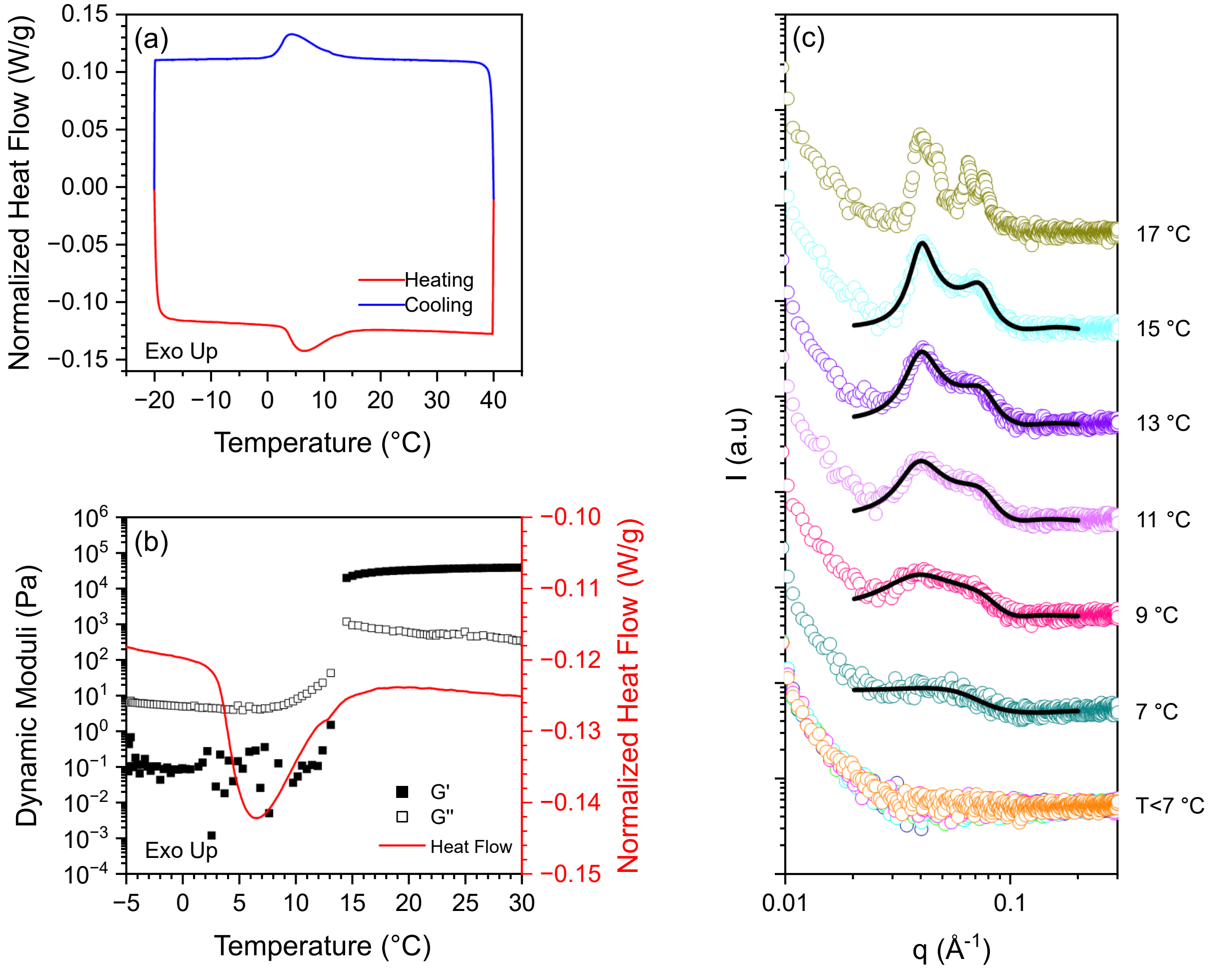

Figure S5. Effect of temperature on the structure and flow behavior of P20EAN: (a) thermal transitions of the mesophase in heating and cooling cycle obtained from DSC, (b) rheological behavior of the mesophase upon increasing the temperature compared with DSC thermograms, and (c) scattering of the sample at different temperatures obtained from SAXS.

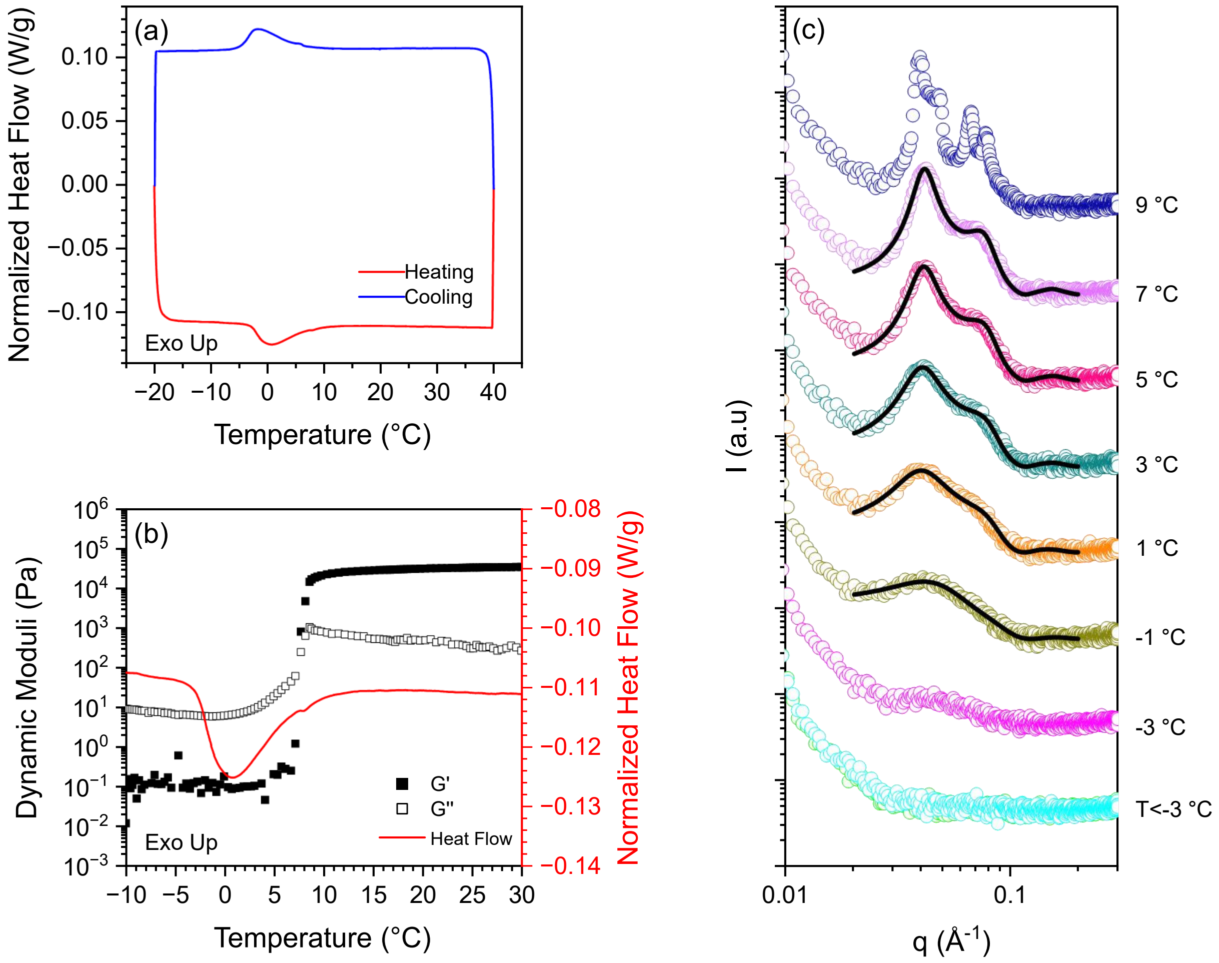

Figure S6. Effect of temperature on the structure and flow behavior of P40EAN: (a) thermal transitions of the mesophase in heating and cooling cycle obtained from DSC, (b) rheological behavior of the mesophase upon increasing the temperature compared with DSC thermograms, and (c) scattering of the sample at different temperatures obtained from SAXS.

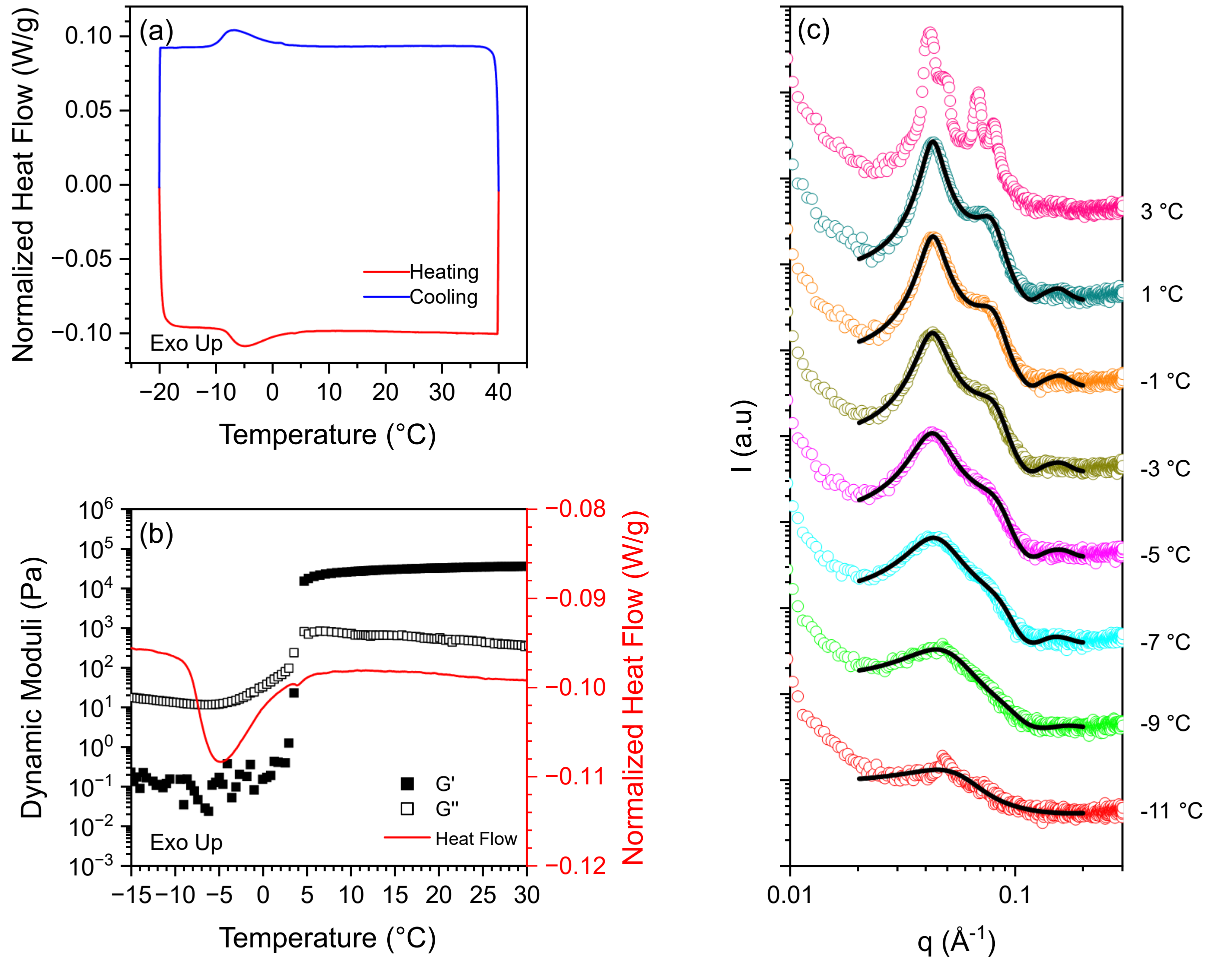

Figure S7. Effect of temperature on the structure and flow behavior of P60EAN: (a) thermal transitions of the mesophase in heating and cooling cycle obtained from DSC, (b) rheological behavior of the mesophase upon increasing the temperature compared with DSC thermograms, and (c) scattering of the sample at different temperatures obtained from SAXS.

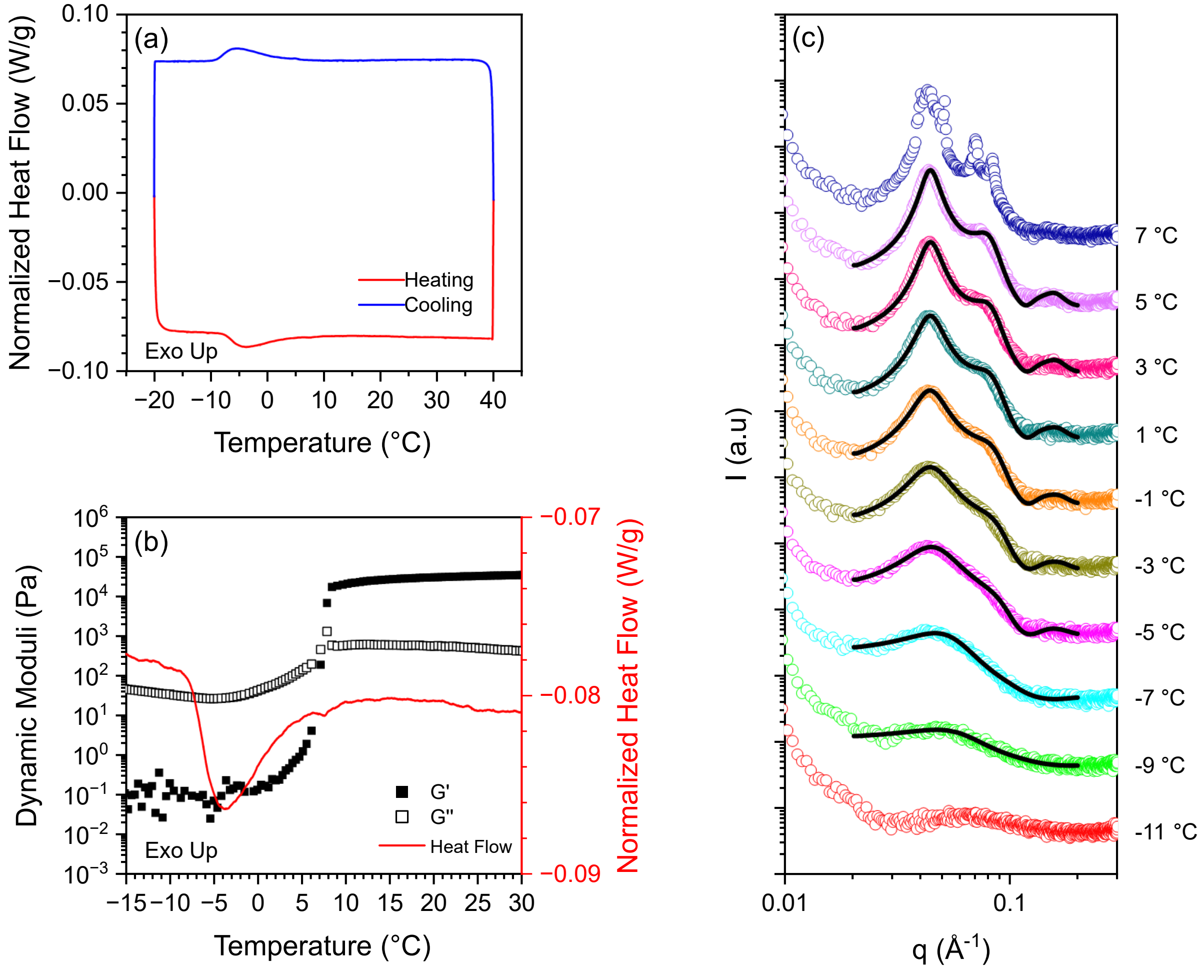

Figure S8. Effect of temperature on the structure and flow behavior of P80EAN: (a) thermal transitions of the mesophase in heating and cooling cycle obtained from DSC, (b) rheological behavior of the mesophase upon increasing the temperature compared with DSC thermograms, and (c) scattering of the sample at different temperatures obtained from SAXS.

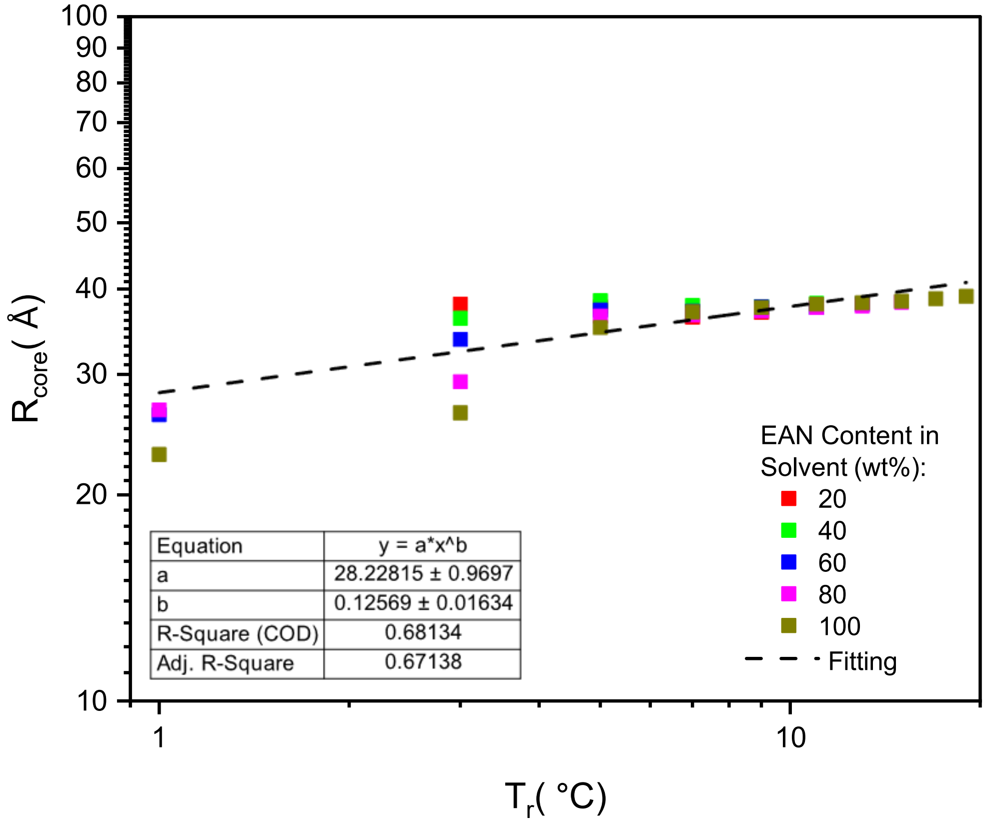

Figure S9. Fitting of R_{core} without considering the solvent effect.

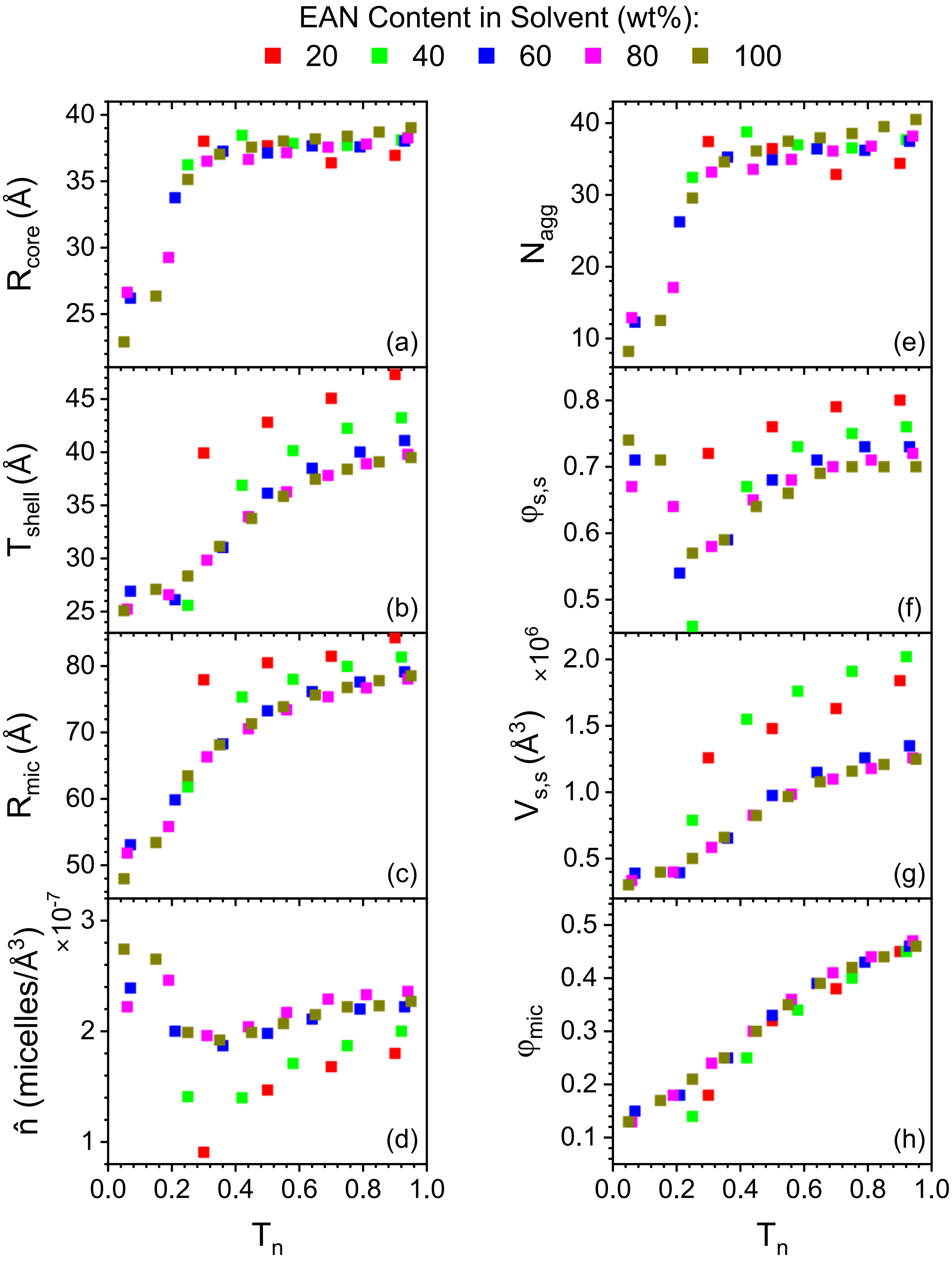

Figure S10. Changes in (a) core radius, R_{core}, (b) shell thickness, T_{shell}, (c) micelle radius, R_{mic}, (d) number of micelles per unit volume, (e) aggregation number, N_{agg}, (f) volume fraction of solvent in shell, $\varphi_{s,s}$, (g) volume of solvent in the shell, and (h) volume fraction of micelles in the system, φ_{mic}, vs T_n upon heating from T_{mic} to T_{gel}. Values are obtained by fitting a core-shell form factor with hard sphere structure factor on the SAXS data.

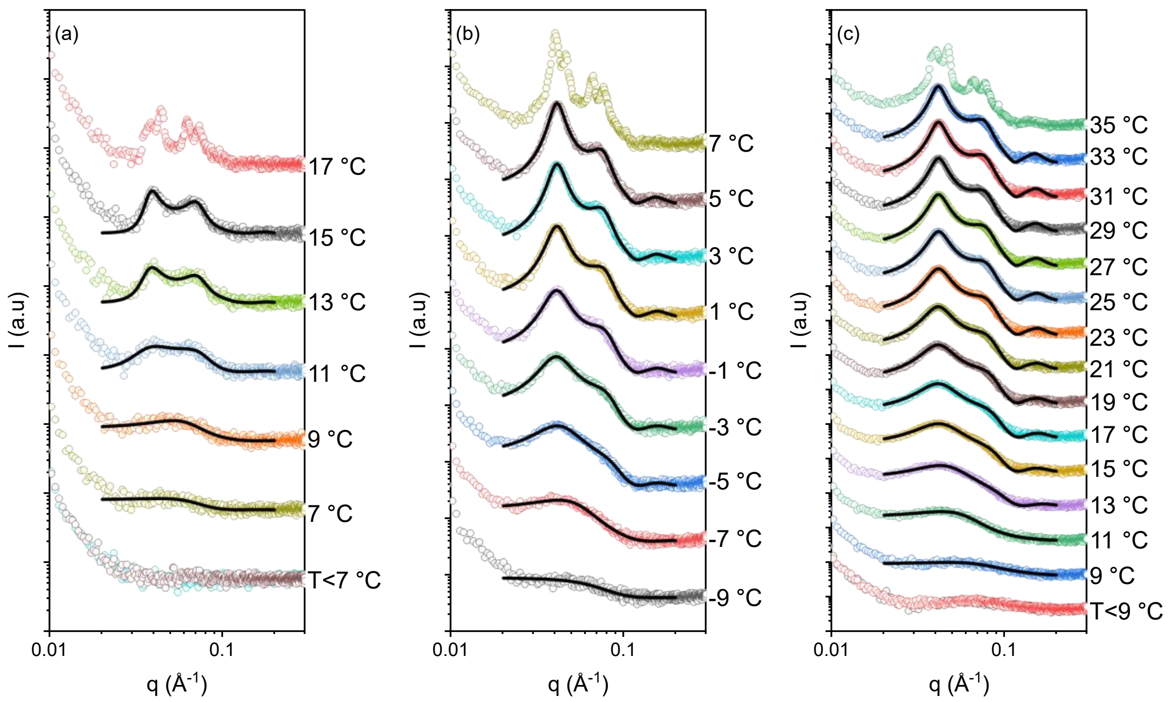

Figure S11. Scattering of the samples with 20 wt% P407 and (a) 20 wt% EAN, (b) 60 wt% EAN, and (c) 100 wt% EAN in the solvent at different temperatures obtained from SAXS.

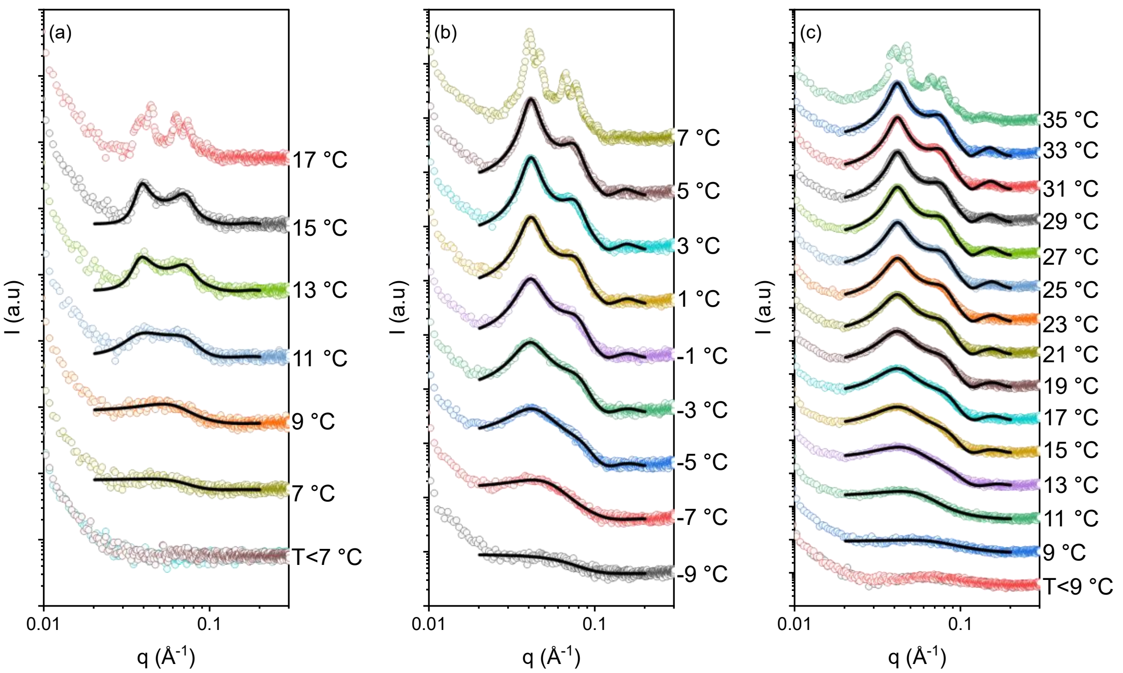

Figure S12. Scattering of the samples with 22 wt% P407 and (a) 20 wt% EAN, (b) 60 wt% EAN, and (c) 100 wt% EAN in the solvent at different temperatures obtained from SAXS.

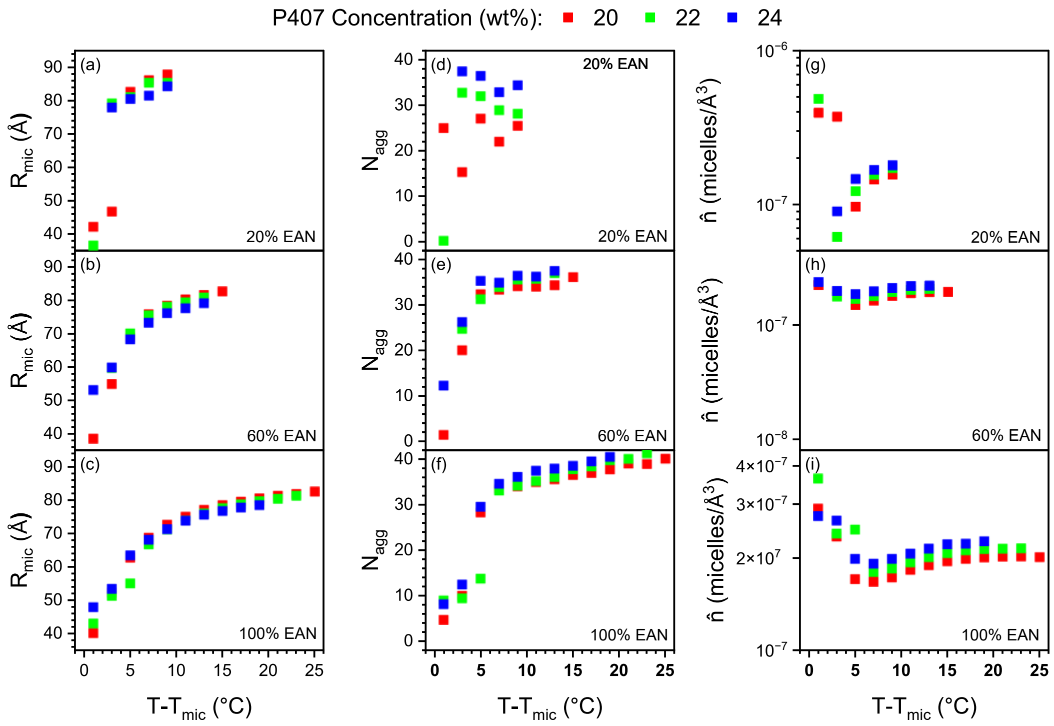

Figure S13. Changes in micelle radius, R_{mic}, aggregation number, N_{agg}, and number density of micelles, $\hat{n}$, of the samples with different P407 concentration and different EAN content in the solvent. Values are obtained by fitting a core-shell form factor with hard sphere structure factor on the SAXS data.

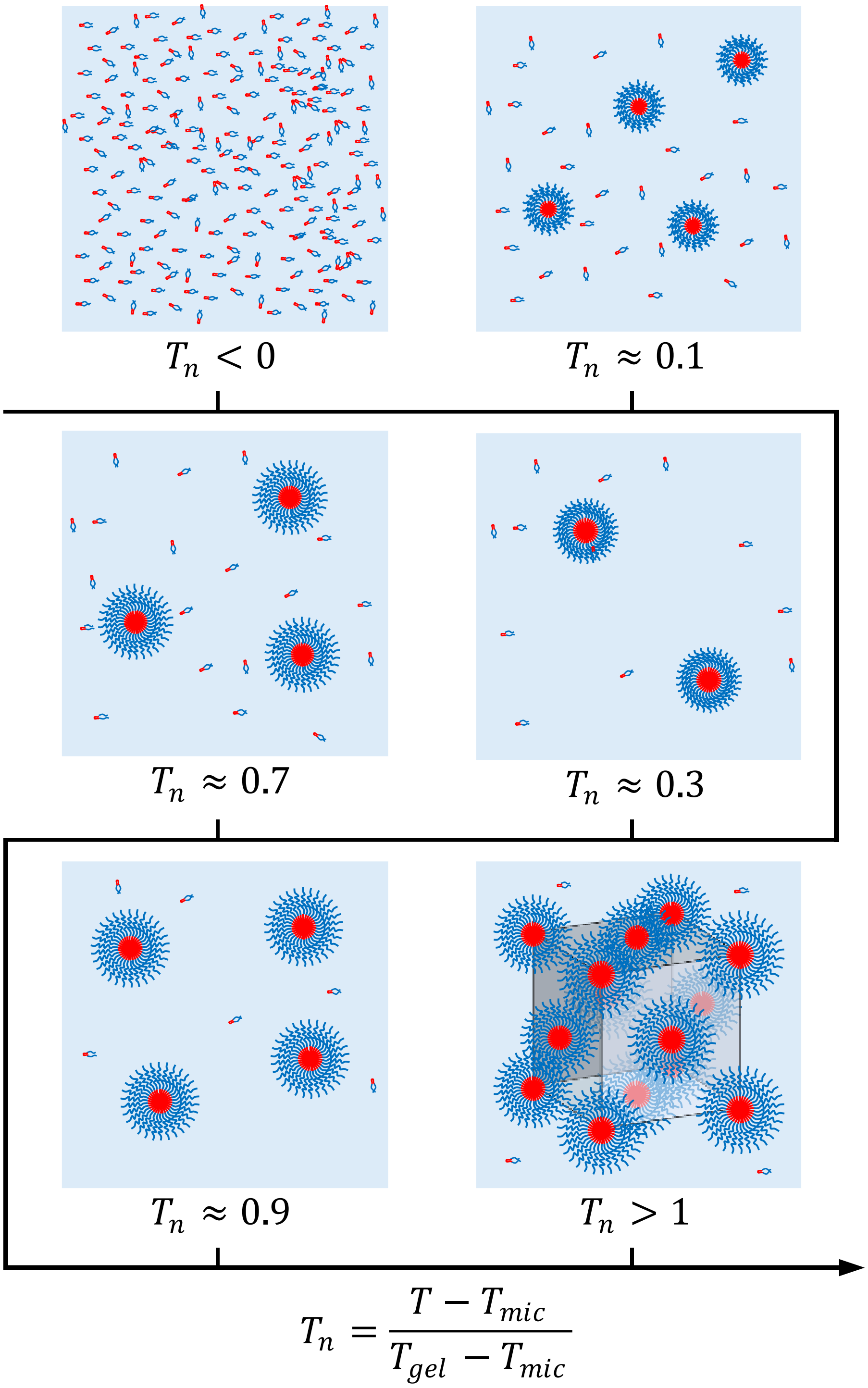

Figure S14. Micellization and self-assembly mechanism of P407 in mixtures of water and EAN upon heating from temperatures below T_{mic} ($T_n < 0$) to above T_{gel} ($T_n > 1$). Size of the unimers are magnified for visualization purposes. Ratios of R_{core} and T_{shell} and number of the micelles in the middle row follow the mechanism proposed here.

Table S1. Sample matrix

Sample Code	EAN in Solvent (wt%)	Weight faction (wt%)			Mol Fraction (mol%)		
		F127	Water	EAN	F127	Water	EAN
P0EAN	0	24	76	0	0.05	99.95	0.00
P20EAN	20	24	60.8	15.2	0.05	95.95	3.99
P40EAN	40	24	45.6	30.4	0.07	89.95	9.98
P60EAN	60	24	30.4	45.6	0.09	79.94	19.97
P80EAN	80	24	15.2	60.8	0.14	59.94	39.92
P100EAN	100	24	0	76	0.27	0.00	99.73

Table S2. Data obtained from analysis of P20EAN SAXS data using spherical core-shell form factor and hard sphere structure factor

T (℃)	T_r (℃)	T_n	R_{core} (Å)	T_{shell} (Å)	R_{mic} (Å)	N_{agg}	$\varphi_{s,s}$	$V_{s,s}$ (10^6Å^3)	φ_{mic}	n ($10^{-7}mic$ /Å^3)
9	3	0.3	37.99	39.92	77.91	37.41	0.72	1.26	0.18	0.91
11	5	0.5	37.65	42.80	80.45	36.43	0.76	1.48	0.32	1.47
13	7	0.7	36.37	45.07	81.44	32.84	0.79	1.63	0.38	1.68
15	9	0.9	36.93	47.30	84.23	34.38	0.80	1.84	0.45	1.80

Table S3. Data obtained from analysis of P40EAN SAXS data using spherical core-shell form factor and hard sphere structure factor

$\boldsymbol{T}$ (℃)	$\boldsymbol{T_r}$ (℃)	$\boldsymbol{T_n}$	$\boldsymbol{R_{core}}$ (Å)	$\boldsymbol{T_{shell}}$ (Å)	$\boldsymbol{R_{mic}}$ (Å)	$\boldsymbol{N_{agg}}$	$\varphi_{s,s}$	$V_{s,s}$ ($\mathbf{10^6}$Å^3)	φ_{mic}	$\boldsymbol{n}$ ($\mathbf{10^{-7}}\boldsymbol{mic}$ /Å^3)
-1	3	0.25	36.22	25.60	61.82	32.43	0.46	0.79	0.14	1.36
1	5	0.42	38.44	36.88	75.32	38.77	0.67	1.55	0.25	1.40
3	7	0.58	37.83	40.14	77.97	36.95	0.73	1.76	0.34	1.71
5	9	0.75	37.68	42.24	79.92	36.53	0.75	1.91	0.40	1.87
7	11	0.92	38.08	43.24	81.32	37.69	0.76	2.02	0.45	2.00

Table S4. Data obtained from analysis of P60EAN SAXS data using spherical core-shell form factor and hard sphere structure factor

$\boldsymbol{T}$ (℃)	$\boldsymbol{T_r}$ (℃)	$\boldsymbol{T_n}$	$\boldsymbol{R_{core}}$ (Å)	$\boldsymbol{T_{shell}}$ (Å)	$\boldsymbol{R_{mic}}$ (Å)	$\boldsymbol{N_{agg}}$	$\varphi_{s,s}$	$V_{s,s}$ ($\mathbf{10^6}$Å$^\mathbf{3}$)	φ_{mic}	$\boldsymbol{n}$ ($\mathbf{10^{-7}}\boldsymbol{mic}$/Å$^\mathbf{3}$)
-11	1	0.07	26.21	26.93	53.14	12.29	0.71	0.39	0.15	2.39
-9	3	0.21	33.75	26.13	59.88	26.24	0.54	0.40	0.18	2.00
-7	5	0.36	37.24	31.04	68.28	35.25	0.59	0.66	0.25	1.87
-5	7	0.50	37.10	36.14	73.24	34.86	0.68	0.98	0.33	1.98
-3	9	0.64	37.64	38.48	76.12	36.40	0.71	1.15	0.39	2.11
-1	11	0.79	37.57	40.02	77.59	36.20	0.73	1.26	0.43	2.20
1	13	0.93	38.00	41.10	79.10	37.46	0.73	1.35	0.46	2.22

Table S5. Data obtained from analysis of P80EAN SAXS data using spherical core-shell form factor and hard sphere structure factor

T (℃)	T_r (℃)	T_n	R_{core} (Å)	T_{shell} (Å)	R_{mic} (Å)	N_{agg}	$\varphi_{s,s}$	$V_{s,s}$ (10^6Å^3)	φ_{mic}	n ($10^{-7}mic$ /Å^3)
-9	1	0.06	26.64	25.25	51.89	12.91	0.67	0.34	0.13	2.22
-7	3	0.19	29.27	26.60	55.87	17.12	0.64	0.40	0.18	2.46
-5	5	0.31	36.49	29.85	66.34	33.17	0.58	0.59	0.24	1.96
-3	7	0.44	36.63	33.93	70.56	33.55	0.65	0.83	0.30	2.04
-1	9	0.56	37.13	36.27	73.40	34.94	0.68	0.99	0.36	2.17
1	11	0.69	37.54	37.82	75.36	36.11	0.70	1.10	0.41	2.29
3	13	0.81	37.77	38.91	76.68	36.78	0.71	1.18	0.44	2.33
5	15	0.94	38.24	39.79	78.03	38.17	0.72	1.26	0.47	2.36

Table S6. Data obtained from analysis of P100EAN SAXS data using spherical core-shell form factor and hard sphere structure factor

$\boldsymbol{T}$ (℃)	$\boldsymbol{T_r}$ (℃)	$\boldsymbol{T_n}$	$\boldsymbol{R_{core}}$ (Å)	$\boldsymbol{T_{shell}}$ (Å)	$\boldsymbol{R_{mic}}$ (Å)	$\boldsymbol{N_{agg}}$	$\varphi_{s,s}$	$V_{s,s}$ ($\mathbf{10^6}$Å^3)	φ_{mic}	$\boldsymbol{n}$ ($\mathbf{10^{-7}} \boldsymbol{mic}$ /Å^3)
7	1	0.05	22.92	25.11	48.03	8.22	0.74	0.31	0.13	2.74
9	3	0.15	26.37	27.11	53.48	12.52	0.71	0.40	0.17	2.65
11	5	0.25	35.12	28.36	63.48	29.57	0.57	0.50	0.21	1.99
13	7	0.35	37.01	31.13	68.14	34.60	0.59	0.66	0.25	1.92
15	9	0.45	37.54	33.77	71.31	36.11	0.64	0.83	0.30	1.99
17	11	0.55	38.00	35.85	73.85	37.46	0.66	0.97	0.35	2.07
19	13	0.65	38.16	37.47	75.63	37.93	0.69	1.08	0.39	2.15
21	15	0.75	38.36	38.40	76.76	38.53	0.70	1.16	0.42	2.22
23	17	0.85	38.68	39.09	77.77	39.50	0.70	1.21	0.44	2.23
25	19	0.95	39.00	39.49	78.49	40.49	0.70	1.25	0.46	2.27

Table S7. Data obtained from analysis of samples with 20 wt% P407 and 20 wt% EAN in the solvent using spherical core-shell form factor and hard sphere structure factor

$\boldsymbol{T}$ (°C)	$\boldsymbol{T_r}$ (°C)	$\boldsymbol{T_n}$	$\boldsymbol{R_{mic}}$ (Å)	$\boldsymbol{N_{agg}}$	φ_{mic}	$\boldsymbol{n}$ $(\mathbf{10^{-7}}\boldsymbol{mic}/\text{Å}^{\mathbf{3}})$
7	1	0.10	42.27	24.99	0.12	3.94
9	3	0.30	46.81	15.35	0.16	3.71
11	5	0.50	82.58	27.08	0.23	0.97
13	7	0.70	86.06	22.02	0.39	1.46
15	9	0.90	87.75	25.49	0.45	1.57

Table S8. Data obtained from analysis of samples with 20 wt% P407 and 60 wt% EAN in the solvent using spherical core-shell form factor and hard sphere structure factor

$\boldsymbol{T}$ (℃)	$\boldsymbol{T_r}$ (℃)	$\boldsymbol{T_n}$	$\boldsymbol{R_{mic}}$ (Å)	$\boldsymbol{N_{agg}}$	φ_{mic}	$\boldsymbol{n}$ $(\mathbf{10^{-7}} \boldsymbol{mic}/\text{Å}^{\mathbf{3}})$
-9	1	0.06	38.60	1.47	0.05	2.24
-7	3	0.19	54.94	20.05	0.14	1.98
-5	5	0.31	68.88	32.33	0.21	1.52
-3	7	0.44	75.81	33.32	0.30	1.66
-1	9	0.56	78.36	34.08	0.36	1.81
1	11	0.69	80.24	34.00	0.41	1.91
3	13	0.81	81.54	34.30	0.44	1.95
5	15	0.94	82.62	36.07	0.46	1.96

Table S9. Data obtained from analysis of samples with 20 wt% P407 and 100 wt% EAN in the solvent using spherical core-shell form factor and hard sphere structure factor

T (°C)	T_r (°C)	T_n	R_{mic} (Å)	N_{agg}	φ_{mic}	n $(10^{-7} mic/Å^3)$
9	1	0.04	40.26	4.78	0.08	2.90
11	3	0.12	52.37	10.04	0.14	2.35
13	5	0.19	62.80	28.31	0.18	1.71
15	7	0.27	68.76	33.50	0.23	1.68
17	9	0.35	72.65	34.03	0.28	1.73
19	11	0.42	75.04	34.96	0.32	1.83
21	13	0.50	77.08	35.59	0.36	1.90
23	15	0.58	78.49	36.53	0.40	1.95
25	17	0.65	79.53	36.98	0.42	1.99
27	19	0.73	80.52	37.75	0.44	2.01
29	21	0.81	81.30	39.02	0.46	2.02
31	23	0.88	81.81	38.94	0.46	2.03
33	25	0.96	82.53	40.10	0.47	2.02

Table S10. Data obtained from analysis of samples with 22 wt% P407 and 20 wt% EAN in the solvent using spherical core-shell form factor and hard sphere structure factor

$\boldsymbol{T}$ (℃)	$\boldsymbol{T_r}$ (℃)	$\boldsymbol{T_n}$	$\boldsymbol{R_{mic}}$ (Å)	$\boldsymbol{N_{agg}}$	φ_{mic}	$\boldsymbol{n}$ ($\boldsymbol{10^{-7} mic}$/Å$^{\boldsymbol{3}}$)
5	1	0.10	36.69	0.28	0.10	4.86
7	3	0.30	79.14	32.74	0.13	0.62
9	5	0.50	81.01	31.99	0.27	1.23
11	7	0.70	85.29	28.91	0.41	1.56
13	9	0.90	85.25	28.14	0.45	1.73

Table S11. Data obtained from analysis of samples with 22 wt% P407 and 60 wt% EAN in the solvent using spherical core-shell form factor and hard sphere structure factor

$\boldsymbol{T}$ (℃)	$\boldsymbol{T_r}$ (℃)	$\boldsymbol{T_n}$	$\boldsymbol{R_{mic}}$ (Å)	$\boldsymbol{N_{agg}}$	φ_{mic}	$\boldsymbol{n}$ $(\mathbf{10^{-7}}\boldsymbol{mic}/\text{Å}^{\mathbf{3}})$
-9	3	0.20	59.73	24.72	0.16	1.79
-7	5	0.33	70.06	31.24	0.25	1.72
-5	7	0.47	75.46	33.92	0.32	1.80
-3	9	0.60	78.04	35.55	0.39	1.96
-1	11	0.73	79.37	35.71	0.43	2.05
1	13	0.87	80.82	36.95	0.46	2.08

Table S12. Data obtained from analysis of samples with 22 wt% P407 and 100 wt% EAN in the solvent using spherical core-shell form factor and hard sphere structure factor

T (°C)	T_r (°C)	T_n	R_{mic} (Å)	N_{agg}	φ_{mic}	n $(10^{-7} mic/Å^3)$
5	1	0.04	43.15	9.03	0.122	3.62
7	3	0.13	51.39	9.50	0.14	2.40
9	5	0.21	55.12	13.80	0.17	2.48
11	7	0.29	66.78	33.11	0.23	1.80
13	9	0.38	71.20	34.20	0.28	1.85
15	11	0.46	73.90	35.19	0.33	1.94
17	13	0.54	76.01	36.08	0.37	2.02
19	15	0.63	77.60	37.79	0.41	2.08
21	17	0.71	78.72	38.40	0.43	2.12
23	19	0.79	79.67	39.76	0.45	2.14
25	21	0.88	80.38	40.05	0.47	2.15
27	23	0.96	81.29	41.27	0.48	2.15
